# Probing corporeal awareness in women through virtual reality induction of embreathment illusion

**DOI:** 10.1038/s41598-024-59766-1

**Published:** 2024-04-23

**Authors:** Chiara Cantoni, Andrea Salaris, Alessandro Monti, Giuseppina Porciello, Salvatore Maria Aglioti

**Affiliations:** 1 Department of Psychology, Sapienza University of Rome, 00185 Rome, Italy; 2 IRCCS Fondazione Santa Lucia, 00179 Rome, Italy; 3 CLN2S@Sapienza, Istituto Italiano di Tecnologia, Sapienza University Rome, 00161 Rome, Italy

**Keywords:** Social neuroscience, Psychology

## Abstract

We capitalized on the respiratory bodily illusion that we discovered in a previous study and called ‘Embreathment’ where we showed that breathing modulates corporeal awareness in men. Despite the relevance of the issue, no such studies are available in women. To bridge this gap, we tested whether the synchronization of avatar-participant respiration patterns influenced females’ bodily awareness. We collected cardiac and respiratory interoceptive measures, administered body (dis)satisfaction questionnaires, and tracked participants’ menstrual cycles via a mobile app. Our approach allowed us to characterize the ‘Embreathment’ illusion in women, and explore the relationships between menstrual cycle, interoception and body image. We found that breathing was as crucial as visual appearance in eliciting feelings of ownership and held greater significance than any other cue with respect to body agency in both women and men. Moreover, a positive correlation between menstrual cycle days and body image concerns, and a negative correlation between interoceptive sensibility and body dissatisfaction were found, confirming that women’s body dissatisfaction arises during the last days of menstrual cycle and is associated with interoception. These findings have potential implications for corporeal awareness alterations in clinical conditions like eating disorders and schizophrenia.

## Introduction

By integrating signals coming from internal and external senses, the brain builds and maintains the feeling of corporeal awareness, namely the feeling that the body, and body parts, belong to oneself (i.e., body ownership) and that the body acts according to one's will (i.e., body agency) and occupies a specific position in space (i.e., body location)^1–3^. Efferent and afferent information jointly contribute to build the core of our bodily awareness^4,5^. This integration arises in multimodal areas of right hemisphere, such as right parietal cortex and temporal parietal junction^6,7^. To date, most of the research on bodily awareness has focused on the use of external objects, such as the rubber hand, to induce several types of bodily illusions. In the last few years, to study the different aspects of corporeal awareness, in particular the sense of body ownership, researchers use immersive virtual reality, a tool which can create the illusion that a virtual body (i.e., an avatar) or a part of a virtual body (i.e., a virtual hand), belongs to us exactly as if it was our own^8–13^. Specifically, studies on bodily illusions (e.g., the rubber hand illusion^14^; the full-body illusion^9^; the enfacement illusion^10^, and the illusory experience of being touched^15,16^) measured how bodily awareness changes as a result of experimental manipulations involving multisensory stimulation protocols. Those protocols, conducted in both real experimental and virtual reality contexts, primarily focused on external cues (mainly visual and tactile) and have been proven to be effective for both research and clinical purposes^17^.

However, bodily illusions can also be induced through interoceptive signals, i.e., signals coming from our internal organs^18,19^ and interoceptive signals significantly contribute to bodily awareness^15,16,20–25^ (but see^26^ for different results). Among internal signals, breathing provides us with feedback on our physiological and health status^27^, and directly influences brain activity (for a recent review see^28^). Breathing has a strong interoceptive component. Indeed, information about lung volume changes is transmitted by the Piezo-2 receptors that are involved in many interoceptive functions^29,30^. Similarly to other visceral functions, breathing involves a complex flow from the lung to the brain of both interoceptive and exteroceptive signals^31,32^. However, unlike other visceral processes, breathing uniquely incorporates motor commands originating from both a brainstem driven automatic control and cortical areas driven voluntary control^33–35^. Importantly, the specific contributions of respiratory afferents and motor commands to the respiratory aspect of corporeal awareness remain unclear. Crucial to the present research, studies indicate that breathing-based visual feedback significantly increases the embodiment of a virtual avatar^36,37^. In particular, our research group developed a novel virtual reality-based paradigm in which we manipulated the congruency of the breathing between the avatar and the participant (i.e., the avatar could breathe in phase, i.e., congruent, or in anti-phase, i.e., incongruent, with respect to the participant), the visual appearance of the avatar (which could be human-like, i.e., congruent, or wooden-like, i.e., incongruent) and the perspective (i.e., the participant could see the avatar from the first, i.e., congruent, or third person, i.e., incongruent, perspective), in order to explore which aspect of bodily awareness (i.e., ownership, agency and location) was most impacted by the illusion. Through this paradigm, we showcased that breathing affects bodily awareness in a sample of male participants and that the strength of this effect depended on participants’ ability to be aware of visceral, interoceptive signals^22^. Specifically, our results on males indicate that, in determining participants’ sense of bodily ownership, breathing is nearly as important as the physical (visual) resemblance between the participants’ body and an embodied avatar. Moreover, breathing does not play a role in determining where the body is located in the space, and it is stronger than both physical resemblance and perspective in determining the sense of agency. Finally, in line with previous literature^38,39^, these effects^22^ are stronger in participants having a lower knowledge of their cardiac and respiratory signals (i.e., lower interoception). What remains unclear is whether the breathing cues provided by our ecologically plausible environment can also modulate corporeal awareness in women.

To address this outstanding issue, the present study aims to investigate the role of breathing in shaping women’s corporeal awareness. Specifically, besides testing which of the experimental manipulations of this paradigm (i.e., visual appearance, perspective and breathing of the virtual agent) is the most effective in eliciting the embreathment illusion, we aim to investigate the relationship between corporeal awareness, body dissatisfaction, menstrual cycle, and interoception in a sample of young healthy females.

Concerning the importance of visual appearance and body image, literature shows that women often perceive their body image as more unsatisfactory compared to men^40–42^. We consider this as a potential factor that could influence the strength of the illusion experienced by women. Indeed, existing studies consistently demonstrate greater body dissatisfaction in women compared to men^40,42–48^. This discrepancy may lead women to place more emphasis on the visual appearance of the avatar than their male counterparts. Importantly, individuals with higher body dissatisfaction also exhibit increased bodily self-plasticity as evidenced by heightened susceptibility to bodily illusions^11^. Body dissatisfaction may also lead to the development of eating disorders, such as anorexia nervosa^11,49^, and this condition is more prevalent in females than in males (see for example^50^ for a recent review). Additionally, empirical evidence suggests that interoception is related to body dissatisfaction^51^, and that body dissatisfaction changes not only during critical periods in women, such as pregnancy^52^, but also alongside the menstrual cycle^53,54^. In particular, research has found that the premenstrual phase is characterized by higher levels of body dissatisfaction compared to the menstrual phase^55^. This might be due to the fact that during the premenstrual phase, women experience a distortion of the perception of their body image, which results, for example, in perceiving an increase in body size^56,57^, an overestimation of waist size^58^ or to a higher focus on unattractive bodily parts^59^. This distortion might be caused by higher levels of progesterone during the premenstrual phase, which are proven to cause higher body image concerns^60^.

Literature shows that women with high body dissatisfaction, such as women suffering from eating disorders, exhibit a more plastic representation of the bodily self^61^. Considering that women typically experience heightened body dissatisfaction during the premenstrual phase^55^ an increased malleability in body representations is reported during this period. Consequently, they might be more susceptible to experiencing the illusion of Embreathment. Regarding the impact of the breath on the strength of the illusion, we anticipate it will have a substantial impact, as breath serves as a potent interoceptive pathway in both males and females. However, we hypothesise that its effects may differ between women and men due to variations in their breathing patterns (i.e., greater contribution of inspiratory rib cage muscles in females than males) and to morphological differences in the respiratory system (i.e., males’ lungs are bigger in absolute volume and in volume variations) in the two sexes^62,63^.

Concerning the perspective manipulation, we expect it to be a strong feature in both samples due to its importance in inducing a coherent body image of the self^2^.

Thus, we expect not only to replicate our prior findings in women^22^, but also to provide a fine-grained picture of which one of the experimental manipulations affects more corporal awareness components (i.e., sense of body ownership, agency, and location). We also expect to map the relationship between interoception and specific features of female body experience like menstrual cycle (measured through a Period Tracker App) and body image dissatisfaction (measured via self-report questionnaires).

We predict to replicate the results found in males with stronger effects of the embreathment illusion in women with low interoceptive abilities. Since women are less accurate than men in perceiving their interoceptive signals^64^, we also expect a higher embreathment illusion in women compared to men. Finally, we hypothesize that: (i) body (dis)satisfaction will increase along the menstrual cycle; (ii) interoception will negatively correlate with body (dis)satisfaction, and (iii) interoception will decrease along the menstrual cycle when a higher body dissatisfaction is expected.

## Materials and methods

### Participants

Thirty-five healthy female volunteers participated in the study after providing their written informed consent. Data from two participants were excluded from the analyses due to technical issues with the virtual reality system. The final sample, in line with Monti and collaborators^22^, included thirty-three participants (mean = 24.57 ± 3.4 yr., range = 19–35 yr.). Inclusion criteria required for all participants to have a regular menstrual cycle, and not to take any hormonal contraceptives or drugs that could alter the regularity of their menstrual cycle. Moreover, participants had no history of psychiatric, neurological, cardiac, or respiratory disorders.

To compare our results with previous findings, published by Monti and collaborators^22^ we compared the present data with data collected from 32 young males (mean age = 22.31 yr, age range = 19–33 yr). The mean age of our women sample was selected to be comparable to that of the original study on the Embreathment illusion conducted in men by Monti and colleagues (2020)^22^.

The experimental protocol was reviewed and approved by the local ethics committee at the Fondazione Santa Lucia Research Hospital (Protocol number: CE/PROG.944). The study was performed in accordance with the ethical standards of the 2013 Declaration of Helsinki. Participants were recruited outside the laboratory from a subject pool of local university students. All of them were naïve to the research purpose, and none received any monetary compensation.

### Procedure

**Figure 1:**
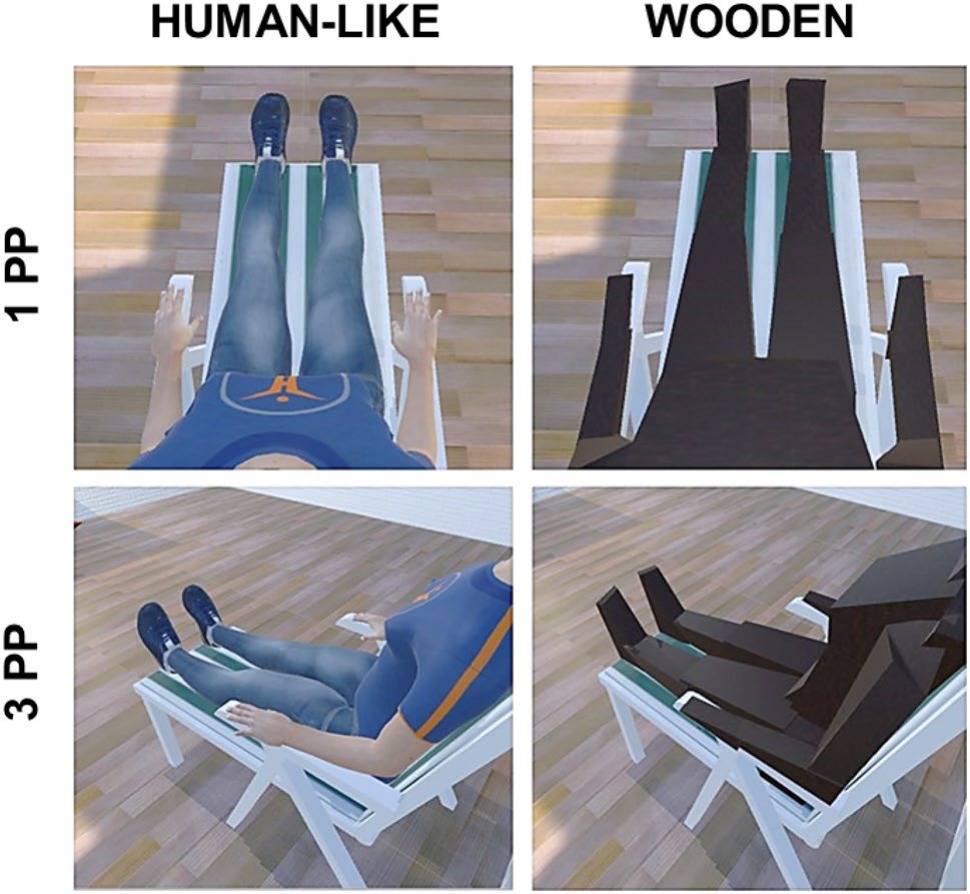
Example of virtual scenarios. Upper-left: human-like avatar shown in a first-person perspective (1PP); Upper-right: wooden avatar shown in a first-person perspective (1PP); Lower-left: human-like avatar shown in a third-person perspective (3PP); Lower-right: wooden avatar shown in a third-person perspective (3PP). A video of the experimental set-up is available here: https://youtu.be/RRPGHmVO3vU.

Participants underwent an immersive virtual reality experience adapted from an experimental paradigm previously conceived by Monti and colleagues^22^, consisting of eight experimental conditions. The order of the experimental conditions was counterbalanced across participants. To accommodate the variations in participants' body dimensions at the outset of the procedure, we requested participants to select the avatar that best resembled their own from a set of options with ten different body dimensions. In each block, participants observed a virtual scenario in which an avatar could have a human or a wooden appearance, was seen in a first-person or third-person perspective, and could breathe in phase with the participant or follow an opposite pattern, thanks to a customized immersive virtual reality setup seen through an HTC VIVE Head Mounted Display (HMD) (HTC Corp.; see Fig. 1 and Video S1). Condition 1 was the most incongruent one (i.e., a wooden-like avatar seen in a third-person perspective and breathing in anti-phase with the participant), Condition 8 was the most congruent (i.e., a human-like avatar seen in first perspective and breathing in phase with the participant), while Conditions 2–7 showed all possible intermediate combinations of congruent and incongruent bodily signals (Table S1). At the end of each block, participants answered a five-item questionnaire to assess the degree to which they experienced feelings of embodiment (Table S3) using visual analogue scales (VAS) ranging from 0 (“*I did not experience that feeling at all*”) to 100 (“*I perceived a strong feeling of that sort*”). We employed the same 5-item embodiment questionnaire previously used by Monti and colleagues^22,23^. Despite being explicit, this questionnaire remains the most widely used tool for gauging embodiment, as highlighted in a recent review by Guy et al. (2023)^65^. Each condition lasted two minutes (for a more detailed description of the apparatus used and the experimental procedure see *Supplementary Materials*).

After the embodiment procedure, participants completed a cardiac interoceptive accuracy task, namely the “Heartbeat Counting Task”^66^, and a respiratory interoceptive accuracy task, namely the “Pneumoception Task”^22^. In the cardiac accuracy task, participants were asked to count their heartbeats during four different randomized time intervals (25 s, 35 s, 45 s, 100 s) while undergoing a bipolar lead II ECG recording. In the respiratory task, each subject was asked to listen to a series of twenty-six unlabelled breathing soundtracks and report if they were “self-tracks” or “non-self-tracks”. Unbeknownst to the participants, half of the tracks contained their breathing sounds, whereas the other half was taken from a frequency- and amplitude-matched control female individual. Finally, participants completed a series of questionnaires: the Italian version of the Multidimensional Assessment of Interoceptive Awareness-2nd version (MAIA-II) (adapted from^67^) to assess their interoceptive sensibility^68^; the Silhouettes Rating Scale Test^69^ for measuring their body image (dis)satisfaction; and the Body Image Concerns subscale of the Body Uneasiness Test^70^ to gauge their concerns for body image. The regularity of participants’ menstrual cycle was assessed by means of the Italian version of the “Flo Ovulation & Period Tracker” app (Flo Health, Inc). We considered the menstrual cycle as a continuum instead of dividing it into premenstrual and non-premenstrual phases. See *Supplementary Materials* for further details about the questionnaires.

## Results

### Self-reported measures of the embreathment illusion

#### Data analysis

To test if the effects of the embreathment illusion found in males by Monti and colleagues^22^ were replicated in a female sample, we built three different linear mixed-effects models. Specifically, we aimed to verify if changes in breathing, visual appearance, and perspective of the virtual body influenced the perception of body ownership, agency, and location. Each model featured the VAS scores of perceived body ownership, agency, and location, respectively, as its dependent variables, and tested the three experimental manipulations as fixed effects (breathing: phase vs. antiphase; perspective: first vs. third; visual appearance: human-like vs. wooden-like). Moreover, the models included interoceptive sensibility (computed, as in Monti and colleagues^22^, as the mean between three subscales of interest of MAIA-II^67^: “Noticing”, “Attention regulation”, and “Body listening”) and interoceptive accuracy (calculated as the mean between scores in the Heartbeat Counting Task^66^ and Pneumoception Task^22^). These interoceptive measures were tested for interactions with fixed effects. The models had a by-subject random intercept.

#### Results

##### Sense of body ownership

**Figure 2:**
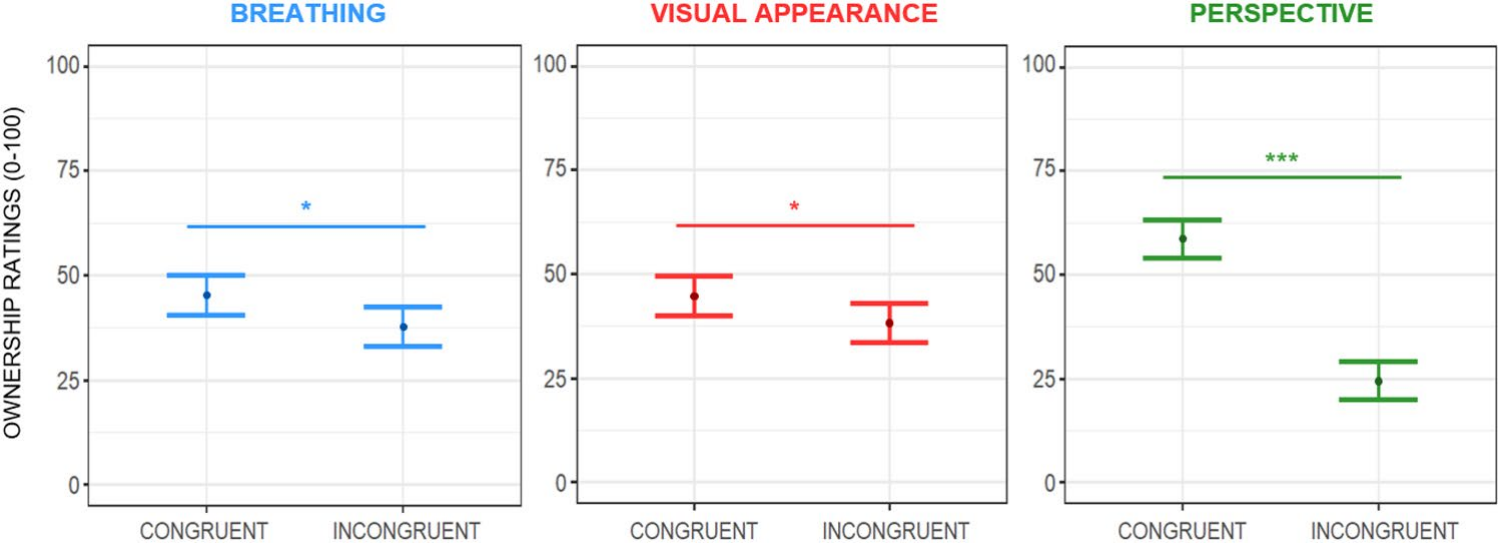
Estimated main effects of breathing, visual appearance, and perspective on perceived ownership during the ‘Embreathment illusion’ procedure on a female sample. Dots represent the estimated effects of the means, while the bars represent the standard errors. Ratings of perceived body ownership increased when the avatar breathed in phase with the participant (left panel), when it was a humanoid-like avatar (middle panel), and when it was shown in first-person perspective (right panel); Significance: ‘***’*p* ≤ .001; ‘*’*p* < .05.

The linear mixed effects model built to parse the subjective ratings of body ownership showed a main effect of breathing (F(1,210) = 5.77; *p* = 0.017) (see Fig. 2, left panel), meaning that participants felt that the virtual body belonged to them more when it breathed congruently with them than when it breathed incongruently, i.e., in anti-phase. This is reflected in increased sense of ownership scores of ~ 14.27 ± 6.14 VAS points when the avatar breathes in phase with respect to when it breathes in anti-phase. Moreover, we found a main effect of visual appearance (F(1,210) = 4.47; *p* = 0.036) (see Fig. 2, middle panel), with a higher perceived sense of ownership when participants embodied the human-like virtual body (increment of ~ 7.01 ± 6.14 points) relative to the wooden-like virtual body. There was also a statistically significant interaction between the visual appearance of the avatar and interoceptive sensibility (F(1,210) = 8.61; *p* = 0.004), however, post-hoc analysis of this interaction, performed with the *emtrends* function of the *emmeans* package^71^ with Bonferroni-adjusted p values, did not show any significant slope. Lastly, we observed a main effect of perspective (F(1,210) = 122.69; *p* < 0.001; see Fig. 2, right panel) that accounted for an increased feeling of owning the avatar’s body of ~ 37.56 ± 6.14 points when it was shown in first-person perspective compared to the third-person perspective. All the other interaction effects were not significant.

##### Sense of body agency

**Figure 3:**
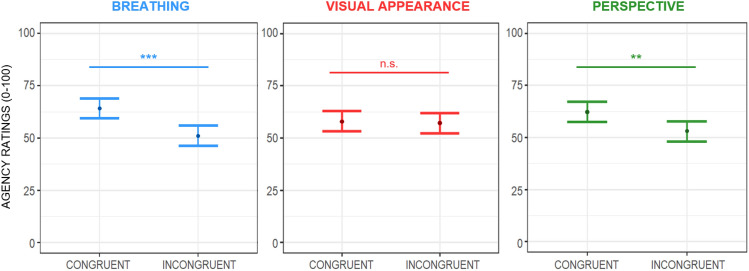
Estimated main effects of breathing, visual appearance, and perspective on perceived body agency during the ‘Embreathment illusion’ in a female sample. Dots represent the estimate effects of the means, while the bars represent the standard errors. Ratings of perceived agency increased when the avatar breathed in phase with the participant (left panel) and when the avatar was shown in first-person perspective (right panel). Significance: ‘***’*p* ≤ .001; ‘**’*p* < .01; ‘n.s.’ non-significant results.

**Figure 4:**
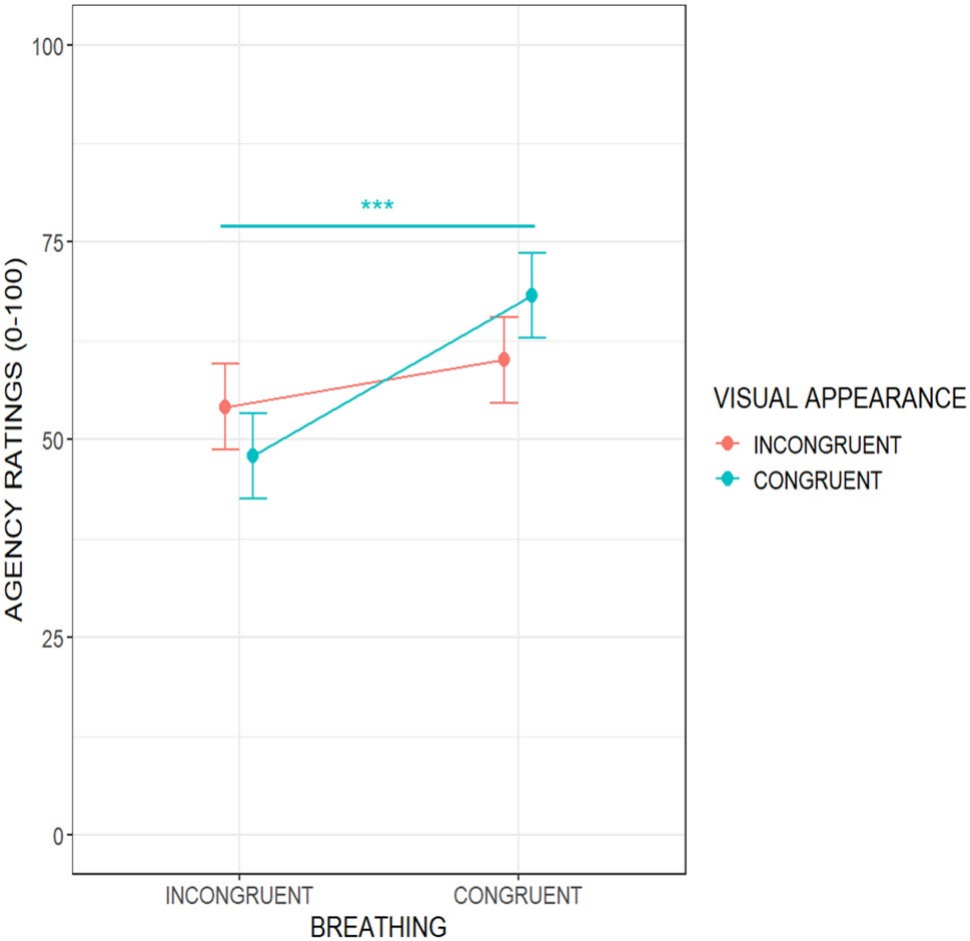
Interaction between breathing and visual appearance on perceived body agency during the ‘Embreathment illusion’ in a female sample. Participants felt they controlled the avatar’s movements more when it was a human-like avatar and it breathed in phase with the participants vs. when it was a human-like avatar, and it breathed with an opposite pattern. Significance: ‘***’*p* ≤ .001.

The linear mixed effects model fitted to test changes over the subjective ratings of perceived body agency showed a main effect of breathing (F(1,210) = 14.65; *p* < 0.001). Participants felt they controlled the avatar’s movements more when it breathed in phase than in anti-phase, showing increased sense of agency scores of ~ 15.81 ± 6.84 points (see Fig. 3, left panel). Moreover, we found a main effect of perspective (F(1,210) = 7.45; *p* = 0.007). Scores in agency rose by ~ 21.04 ± 6.84 VAS points when participants embodied the avatar in the first-person perspective compared to the third-person perspective (see Fig. 3, right panel). We also found a statistically significant interaction between breathing pattern and the avatar’s visual appearance (F(1,210) = 4.41; *p* = 0.037). Post-hoc analysis run using the *emmeans* package^71^ with Bonferroni-adjusted *p*-values revealed that participants felt they controlled the avatar’s movements more when it was a human-like virtual body and it breathed in phase with the participants *vs.* when it was a human-like avatar and it breathed with an opposite pattern (*p* < 0.001) (see Fig. 4). All the other main or interaction effects were not significant.

##### Sense of body location

**Figure 5:**
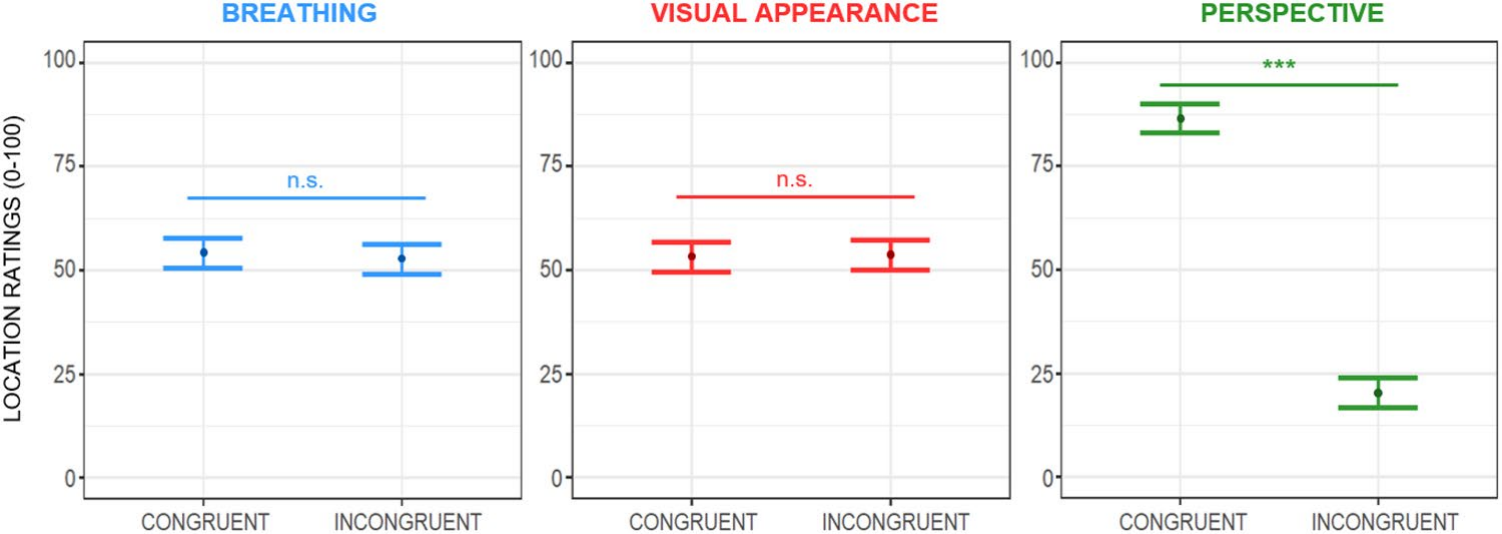
Estimated main effect of breathing, visual appearance, and perspective on perceived body location during the ‘Embreathment illusion’ in a female sample. Dots represent the estimate effects of the means, while the bars represent the standard errors. Ratings of perceived body location increased when the avatar was shown from a first-person perspective (right panel). Significance: ‘***’*p* ≤ .001; ‘n.s.’ non-significant results.

The linear mixed effects model run to assess changes over the subjective ratings of body location revealed a main effect of perspective (F(1,210) = 664.98; *p* < 0.001). Participants reported they felt they occupied the same space of the avatar more with a first-person than a third-person point of view (~ 66.43 ± 5.12 VAS points). The main effect of perspective on the body's perceived location is shown in Fig. 5. All the other main or interaction effects were not significant.

### Sex differences in the embreathment illusion

#### Data analysis

To test sex differences in the embreathment illusion, we built three linear mixed models, one for each component of corporeal awareness (i.e., ownership, agency, and location), following the same procedure described for the female sample, but adding sex as a factor. For these analyses, we combined data collected in the present study with data previously collected by Monti and collaborators using the very same procedures^22^.

#### Results

##### Sex differences in the sense of body ownership

The linear mixed-effects model created to analyse sex differences in body ownership ratings revealed a significant main effect of breathing (F(1,412) = 21.16; *p* ≤ 0.001), indicating that participants felt a stronger sense of ownership over the virtual body when it breathed synchronously with their own breathing compared to when it breathed following an opposite pattern. This is reflected by an increase of approximately 14.36 ± 5.68 VAS points in the sense of ownership when the avatar’s breathing was in-phase with the participant’s breathing. We also found a significant interaction between breathing and participants’ interoceptive accuracy (F(1,412) = 5.87; *p* = 0.016). However, post-hoc analysis of this interaction did not reveal any significant differences. Additionally, we found a significant main effect of visual appearance (F(1,412) = 17.33; *p* < 0.001), with participants reporting a higher perceived sense of ownership when embodying the human-like virtual body (an increment of ~ 7.50 ± 5.68 points relative to the wooden-like virtual body). There was also a statistically significant interaction between the visual appearance of the avatar and interoceptive sensibility (F(1,412) = 5.93; *p* = 0.015). However, post-hoc analysis of this interaction, conducted using the *emtrends* function of the *emmeans* package^71^ with Bonferroni-adjusted *p*-values, did not reveal any significant slope. Furthermore, we observed a significant main effect of perspective (F(1,412) = 311.2; *p* < 0.001): participants’ feeling of owning the avatar’s body increased by approximately 37.89 ± 5.68 points when the avatar was shown in a first-person perspective compared to a third-person perspective. Finally, a significant interaction between the avatar’s perspective and the participant’s interoceptive sensibility emerged (F(1,412) = 13.35; *p* < 0.001). Also in this case, post-hoc analysis did not show any significant result. All the remaining main and interaction effects were not significant. A detailed summary of the results is shown in Table S8 of *Supplementary Materials*. Results from this model confirm all the effects observed and described in detail in the section above (“Self-reported measures of the Embreathment illusion”) where only data from the female sample were analysed.

##### Sex differences in the sense of body agency

The linear mixed effects model used to examine changes in subjective ratings of perceived body agency in males and females revealed a significant main effect of breathing (F(1,412) = 29.16; *p* < 0.001), indicating that participants felt a greater sense of control over the avatar’s movements when it breathed in phase compared to an anti-phase pattern, resulting in an increased sense of agency scores (approximately ~ 16.281 ± 6.26 points). Secondly, a main effect of perspective was found (F(1,412) = 16.25; *p* < 0.001). Participants reported higher agency scores (~ 21.41 ± 6.26 VAS points) when embodying the avatar from a first-person perspective, compared to the third-person perspective. Furthermore, a significant interaction was found between breathing pattern and the avatar’s visual appearance (F(1,412) = 4.71; *p* = 0.030). Post-hoc analysis, conducted using the *emmeans* package^71^ with Bonferroni-adjusted *p*-values, revealed that when the avatar had a human-like virtual body and breathed in phase with the participants, there was a stronger sense of control over its movements, as opposed to when it had a human-like appearance but breathed in an opposite pattern (*p* < 0.001). All the remaining main and interaction effects were not significant. A detailed summary of the results is shown in Table S9 of *Supplementary Materials*. Results from this model confirm all the effects observed and described in detail in the section above (“Self-reported measures of the Embreathment illusion”) where only data from the female sample were analysed.

##### Sex differences in the sense of body location

**Figure 6:**
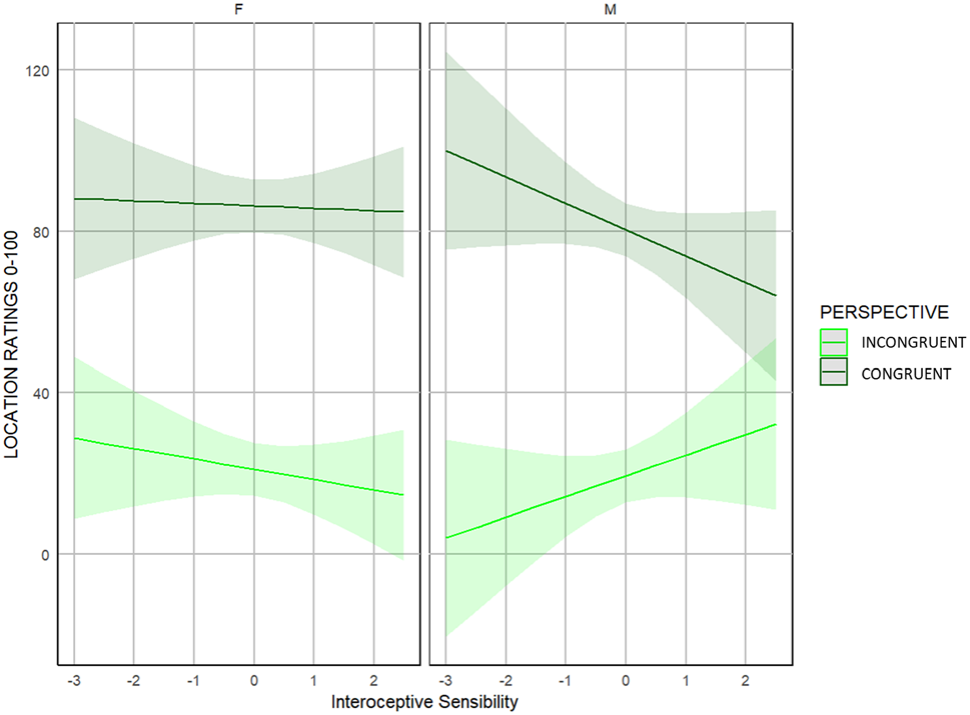
Interaction effect of the avatar’s perspective and interoceptive sensibility on perceived body location during the ‘Embreathment illusion’ in both male and female samples. Ratings of perceived body location differed when the avatar was shown in first-person perspective compared to the third-person perspective in men with lower interoceptive sensibility. This difference smoothed out as interoceptive sensibility increased. The difference between the slopes was not significant in the females. Male sample data were taken from Monti and colleagues’ dataset^22^.

The linear mixed-effects model used to examine changes in the subjective ratings of body location in males and females showed a significant main effect of perspective (F(1,412) = 1246.36; *p* < 0.001). Participants reported feeling a stronger sense of occupying the same space as the avatar when viewing from a first-person perspective compared to a third-person perspective (~ 65.19 ± 5.01 VAS points). We also observed significant interactions of the perspective with participants’ interoceptive accuracy (F(1,412) = 17.52; *p* < 0.001), and participant’s interoceptive sensibility (F(1,412) = 6.29; *p* = 0.012). After running post-hoc analysis, no slope was found to be significant. Finally, we found a triple significant interaction among avatar’s perspective, participants’ interoceptive sensibility and the sex of participants (F(1,412) = 12.36; *p* < 0.001). Post-hoc analysis run using the *emtrends* function of *emmeans* package^71^ showed a significant difference between the slopes predicting location ratings from interoceptive sensibility scores in congruent and incongruent perspective in males (t-ratio: 3.808; *p*-value: 0.001) but not in females (t-ratio: − 0,824; *p*-value:1.00). Specifically, ratings of perceived body location changed depending on interoceptive sensibility in men when the avatar was shown in first-person perspective compared to the third-person perspective: as men's interoceptive sensitivity increases, the difference in body location ratings for the two different perspectives flattened out. This was not the case in the female sample. (Fig. 6). All the remaining main and interaction effects were not significant**.** A detailed summary of the results is shown in Table S10 of *Supplementary Materials*. Results from this model confirm all the effects observed and described in detail in the section above (“Self-reported measures of the Embreathment illusion”) where only data from the female sample were analysed.

### Sex differences in interoception

#### Data analysis

To explore the differences between men and women in interoceptive accuracy (measured with the Heartbeat Counting Task and the Pneumoception task) and interoceptive sensibility (assessed via MAIA-II questionnaire, as we stated above by averaging the three subscales of interest), we ran a series of T-tests.

#### Results

In line with the literature^64^, we found that men had higher scores than women in the Heartbeat Counting Task (t(1,54) = − 2.46, *p* = 0.017) (Figure S1), while no significant difference emerged between the two groups in the Pneumoception Task (t(1,51.6) = 0.495, *p* = 0.623) and in the self-reported measure of interoceptive sensibility (t(1,59.5) = 0.400, *p* = 0.691).

### Relationships between interoception, body image concerns/(dis)satisfaction and the menstrual cycle

#### Data analysis

To test the relationships between the menstrual cycle, interoception and women’s body image concerns/(dis)satisfaction, we ran a series of correlations between self-report measures of body image concerns/(dis)satisfaction (BIC subscale of BUT questionnaire and ratings at the Silhouettes Rating Scale Test, respectively), menstrual cycle days, and interoceptive sensibility (MAIA-II questionnaire). In order to deal with multiple comparisons, Bonferroni correction method^72^ was used.

#### Results

**Figure 7:**
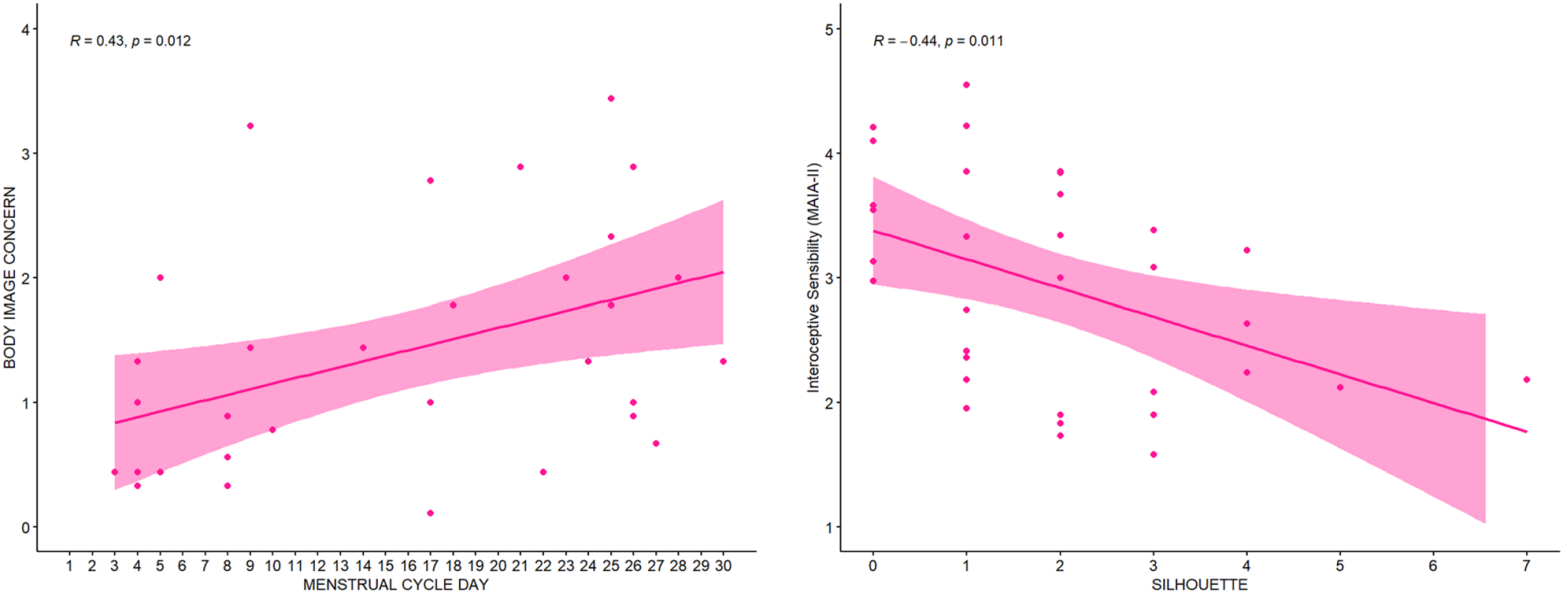
Left panel: positive correlation between self-reported body concerns (measured through the Body Image Concerns, BIC subscale of the BUT questionnaire) and menstrual cycle day (measured through the ‘Leaf’ app). Right panel: negative correlation between interoceptive sensibility (Isen) measured through MAIA-II questionnaire and body image (dis)satisfaction (measured through the Silhouettes Rating Scale Test). The body dissatisfaction index (Silhouettes Rating Scale Test) is calculated by subtracting the value (from 1 to 9) of the ideal body image from the value (from 1 to 9) of the real body image, in absolute value, the higher is the score, the higher is body dissatisfaction. Here only the range of values of the analysed female sample is represented (from 0 to 7 in absolute value).

A correlation between women’s body image concerns, measured through the “Body Image Concern” subscale of the BUT and menstrual cycle day emerged, suggesting that the closer women approach the premenstrual period, the more their body dissatisfaction increases (r(31) = 0.43; pBonf = 0.012), (Fig. 7, left panel). Moreover, we found a negative correlation between women’s body image dissatisfaction, measured through the Silhouettes test, and Interoceptive sensibility, measured via the MAIA-II questionnaire (r(31) = − 0.44, pBonf = 0.011). This result suggests that the more women feel dissatisfied with their own bodies, the lower is their report to be aware of their internal signals (Fig. 7, right panel). All the other correlations did not meet the statistical significance threshold (all *ps* > 0.05).

## Discussion

Expanding on the ‘Embreathment’ illusion paradigm through which we gauged cogent information on the role of breathing on corporeal awareness in men^22^, here we created immersive virtual reality scenarios in which we manipulated the physical appearance, the perspective and breathing pattern of a female avatar. This procedure allowed us to measure the impact of respiratory signals on crucial facets of corporeal awareness, such as the sense of body ownership, agency, and location, in a sample of healthy women.

Specifically, we aimed to explore which experimental manipulation of this paradigm (i.e., visual appearance, perspective and breathing of the virtual agent) was the most effective in eliciting the ‘Embreathment Illusion’^22^.

Moreover, in keeping with the literature we investigated the association between interoceptive abilities, menstrual cycle and body image (dis)satisfaction in women^51–54,59,73^.

We largely replicated the results in men^22^ by finding also in healthy women that breathing significantly influenced the sense of having a body and controlling its movements, thus supporting the view that internal signals of the body play a key role in corporeal awareness.

Specifically, our findings concerning the sense of owning a body are coherent with previous findings on the role of breathing in enhancing this component of body awareness^37,74^. Indeed, Kosuge and colleagues^74^ found that temporal and spatial congruency between an individual’s respiratory rhythm and a mannequin hand increased the feeling of vicarious ownership towards the mannequin hand. In addition to the respiratory channel, in the present study we also found that both avatar appearance and perspective influenced participants' sense of ownership.

Congruent breathing also enhanced participants’ sense of agency, leading to a greater perception of controlling the avatar's movements when the avatar ‘breathed’ in phase with the participants. The sense of controlling the avatar through breathing signals was discussed in two recent reviews^75,76^. Crucially, it appears that different forms of voluntary control over one’s breath may differentially impact the sense of agency^77^. Furthermore, our results of the impact of breathing on the sense of agency are in line with two studies showing how the synchronous flash of the breathing signal onto an avatar silhouette increases the sense of agency and location towards the avatars^33,37^. In the first study by Adler and colleagues^33^, they processed the breathing signal online with a virtual reality experimental software that displayed a halo around an avatar seen from behind in a third-person perspective. The halo transparency was modulated either in synchrony or out of synchrony with the breathing cycle (i.e., the halo became increasingly visible during inspiration and reached a peak at the end of inspiration. The transparency was reset at the beginning of each inspiration). The second study by Allard and colleagues used the very same paradigm as Adler and colleagues by adding an experimental induction of dyspnea^37^. At variance with those two studies, here we used a more ecological manipulation to reproduce breathing signals over the virtual agents, as participants observed human-like or wooden-like avatars breathing in sync/non-synch, thus reproducing our daily life experience of breathing.

In addition to a merely interoceptive component, breathing has an exteroceptive one constituted by the movement performed in the act of breathing. However, it is not clear whether any component plays a dominant role in generating bodily awareness^78^. We acknowledge as a limitation of the present study the fact that we did not have a control condition for teasing apart the specific influence of visual motor cues. This is an important issue that future studies need to take into consideration, for example by creating a control condition which involves visuo-motor signals excluding the interoceptive channel. This can be done, for instance, by asking participants to move body parts which are not associated with any visceral organs, measuring the effect of congruent vs. incongruent feedback on participants’ bodily awareness, and comparing these effects with those triggered by congruent/incongruent respiratory movements.

Regarding the congruence of breathing, our results show that it has an impact on the sense of body ownership (i.e., the feeling that the virtual body/object is mine) and an even higher impact on the sense of body agency (i.e., the feeling to control the movements of the virtual body/object) but not on the sense of body location (i.e., the feeling to be in the same place of the virtual body/object). This could be due to the fact that the sense of occupying the same place as the virtual body/object, as we can see from the results, is highly influenced by perspective, namely by observing the virtual body from the first or third-person perspective. Therefore, in this case the visuo-spatial cue given by the congruent or incongruent perspective may have had a greater influence on the sense of body location than the interoceptive cue provided by the congruent breath of the avatar. This result is coherent with a previous study that showed that if a body is seen in the far extra-personal space (as in our third-person perspective condition), changes in the perception of self-location were not coupled with changes in body ownership^79^.

Moreover, we found that the appearance of the avatar per se does not seem to affect the sense of agency, but agency ratings were influenced by the appearance of the virtual character when also taking into account the congruency of breathing between the avatar and the participant. Indeed, participants showed higher ratings of agency when the human-like avatar breathed synchronously with them. One possible interpretation of this integration of internal and external cues at the basis of the vicarious feeling of agency in women may reside in the greater importance attributed to outer appearance, i.e., body image, that seems to characterize women^51,80^. Recent empirical evidence shows that women tend to focus on their body image more than men ^42,48^. Therefore it is possible that to influence their sense of agency, in addition to the interoceptive cue provided by the congruent breath, congruence with the visual appearance of the avatar is essential in females. Our results also revealed that the interaction effect between visual appearance and breathing appeared when we analyzed the sample of both men and women together. Notably, while this effect did not manifest within the male sample^22^, it potentially originates from the female participants, albeit not achieving statistical significance. Future studies should specifically explore how the avatar's physical appearance alone influences participants’ feeling to control the avatar movements (i.e., sense of agency) in both men and women.

For what concerns the first-person perspective, the present findings confirm that it plays a predominant role in increasing the embodiment of a virtual agent, resulting in a higher sense of body ownership, agency, and location. In fact, participants embodied the virtual agent more when it was presented in first person compared to when it was presented in third person^81^.

Combining the present data and those obtained in our previous report^22^ we have been able to compare the strength of the embreathment illusion in both men and women and to show that breathing shapes bodily awareness equally in both the two groups. Thus, breathing is a salient physiological signal of crucial importance for bodily awareness—especially with respect to the sense of body ownership and agency– regardless of sex. In fact, no main effect of sex emerged in any facets of bodily awareness. An interesting result that emerged when we compared men and women, is the significant interaction between sex, perspective and interoceptive sensibility. This interaction effect is accounted for by the fact that while in women the body location ratings during the perspective manipulation were not influenced by their levels of interoceptive sensibility, the tendency to focus on internal states of the body seems to affect location ratings in men. This interaction effect, in keeping with Prentice and Murphy^82^, indicates that men might rely less on external cues, and therefore have more stable body representations^83^. In fact, in line with a recent review by Prentice and Murphy^82^, we found a significant difference in cardiac interoceptive accuracy task between men and women, with men showing higher scores at the heartbeat counting task. This was not true for interoceptive accuracy measured via the pneumoception task, suggesting that the results in respiratory tasks are less consistent than the cardiac interoceptive tasks. Empirical evidence suggests that individuals may vary in their awareness of different interoceptive channels. For instance, there exists no correlation between cardiac and respiratory interoceptive measures^84^. Hence, it's crucial to measure both channels to capture these peculiarities. For what concerns instead the choice of the task, we decided to measure cardiac interoceptive accuracy via the heartbeat counting task (HBCT), which is the most commonly used interoceptive accuracy task in the cardiac domain. We are aware that the perception of the heart rate can be influenced by several individual physiological and psychological factors^85^ such as personal beliefs^86^, heart rate and heart rate variability^87^, percentage of body fat^88^ and systolic blood pressure^89^. We, therefore, included among the limitations of the present study that an additional cardiac interoceptive accuracy task (e.g., Heartbeat discrimination task) could have been used as a control task.

To investigate whether any specific variable influences women’s interoceptive abilities, we considered the relationship among interoceptive dimensions (accuracy and sensibility), menstrual cycle (day of the menstrual cycle) and women’s negative body image, as inferred from both body image concerns and body dissatisfaction. Studies suggest that negative body image pertains to adverse thoughts and emotions an individual harbours concerning their physical body^90^. This concept encompasses different aspects, such as discontent with one's appearance, vigilant monitoring of the body, feelings of shame related to the body, and preoccupation with one’s body weight^90,91^. Literature shows conflicting evidence regarding the changes in body image during different phases of the menstrual cycle. Some studies suggest that women’s body image perception varies across the menstrual cycle, leading to body image dissatisfaction during the premenstrual phase^53,55,57,58^. Other studies instead indicate that body dissatisfaction is higher during the menstrual and peri-menstrual (premenstrual plus menstrual) phases^54,92^. This might be caused by the fact that during the premenstrual phase women exhibit a distortion of the perception of their body image, which results, for example, in perceiving an increase in body size^56,57^, an overestimation of waist size^58^ or to a higher focus on unattractive body parts^59^. This distortion might be caused by higher levels of progesterone during the premenstrual phase, which are proved to cause higher body image concerns^60^. The body image dissatisfaction during the premenstrual phase could also be explained by interoceptive and physical symptoms such as bloating, breast tenderness and fluid retention^93^. These physical changes are reflected also in premenstrual distress syndrome^94^. Literature shows that in women, premenstrual distress is in fact associated with body image disturbance^95^ and dissatisfaction^96^. However, as stated by Ryan and colleagues^97^, the factors related to this subjective feeling in women during the premenstrual phase remain unclear. Future studies could deepen the role of interoceptive signals during the premenstrual phase, also focusing on women affected by the premenstrual distress syndrome. Here, we found a positive correlation between menstrual cycle days and body image concerns, suggesting that women’s body image dissatisfaction rises when approaching the last days of the menstrual cycle, namely in the premenstrual phase. Undoubtedly, the implementation of an objective hormonal measurement^98^ in our study would have ensured greater precision in defining the hormonal variation according to the phase of the menstrual cycle and, consequently, would have allowed us to have a more precise description of the influence of the menstrual cycle on body image perception. We acknowledge as a limitation the fact we have been unable to collect hormonal data. However, we considered the menstrual cycle as a continuum instead of categorizing women into premenstrual and non-premenstrual phases. This approach allowed us to explore the different nuances of changes in body perception across the entire duration of the menstrual cycle.

It is worth noting that we found a negative correlation between body image (dis)satisfaction, operationalised here as the difference between the perceived and the desired body, and interoceptive sensibility. This aligns with recent empirical evidence and theoretical models proposing that reduced interoception could lead to a situation in which exteroceptive (such as visual, and tactile), and proprioceptive cues play a more prominent role in the awareness of one's body^38,39^. Consequently, this increased focus on external, sensory aspects of the body^51,73^ may contribute to the development of a heightened emphasis on the outward, aesthetic bodily attributes, potentially fostering the emergence of a negative body image^80^. Indeed, individuals displaying body image disturbances and symptoms of eating disorders have been observed to prioritize external sensory cues over internal interoceptive cues in experiments involving body ownership manipulations, such as the rubber hand illusion^61,99^.

As a final remark, we believe that the virtual reality protocol that allowed us to report the “Embreathment Illusion” and establish a clear connection between respiratory cues in both healthy men and women, should be used in further studies involving clinical populations affected by bodily awareness disorders, like schizophrenia^100,101^, as well as body image disorders such as anorexia nervosa^102^, and those aiming to investigate the neural counterpart of the illusion. This virtual reality protocol could be used to gradually enhance the sense of agency and ownership experienced by those patients over healthier versions of their bodies (i.e., the avatars). Moreover, considering that an impaired sense of agency is a feature of psychosis and schizophrenia^103–105^, our virtual reality protocol could be adopted in future studies for gradually rehabilitate the sense of agency in these patients and in patients with eating disorders ^11,106^, as suggested by a review from Koskina and colleagues^107^. Undoubtedly, to be able to adopt this paradigm in contexts such as hospitals or care centers, future studies are needed to enhance its feasibility. For example, using new technologies such as the Ultimate Vive Tracker could greatly improve the paradigm by making the equipment portable, wireless and user-friendly.

This paradigm could also be employed to improve respiratory-impaired conditions like asthma or pulmonary chronic diseases. Indeed, in a recent work, Betka and colleagues found that a virtual reality synchronous visuo-respiratory stimulation improves sensation of breathing control (i.e., sense of body agency) in COVID patients with refractory breathlessness^108^. Finally, this experimental paradigm could inspire future virtual reality rehabilitation protocols similar to the one developed by Blum and colleagues^109^ and be used to improve interoceptive awareness.

### Supplementary Information

Supplementary Information.

## Data Availability

The datasets generated and analysed during the current study and supplementary materials are available on the Open Science Framework repository (https://osf.io/s8xne/).
