# Supplementary material for: Probing corporeal awareness in women through virtual reality induction of embreathment illusion
Source: Sci Rep. 2024 Apr 23;14:9302. doi: 10.1038/s41598-024-59766-1 (PMC11039480; doi:10.1038/s41598-024-59766-1)
Supplement: Supplementary file 1 — Supplementary Information. [file 41598_2024_59766_MOESM1_ESM.pdf]

Probing corporeal awareness in women through virtual reality induction of Embreathment illusion

Chiara Cantoni[1,2*$], Andrea Salaris[1,2*$], Alessandro Monti[1], Giuseppina Porciello[1,2], Salvatore Maria Aglioti[1,2,3]

1. Department of Psychology, Sapienza University of Rome, Rome, 00185, Italy.
2. IRCCS Fondazione Santa Lucia, Rome, 00179, Italy.
3. CLN2S@sapienza, Istituto Italiano di Tecnologia and Sapienza University Rome, Rome, 00161, Italy

* These authors equally contributed to this work and are designated as co-first authors.

[$]Corresponding authors:

Chara Cantoni

Department of Psychology, Sapienza University of Rome

Phone: +39 06 44427 635; Fax: +39 06 44427 635;

E-mail address: chiara.cantoni@uniroma1.it

Andrea Salaris

Department of Psychology, Sapienza University of Rome

Phone: +39 06 44427 635; Fax: +39 06 44427 635;

E-mail address: andrea.salaris@uniroma1.it

Supplemental Data

Supplemental video S1

A video of the experimental setup is available here:

https://youtu.be/RRPGHmVO3vU

Supplemental tables S1-S10

N.	Visual appearance	Breathing	Perspective
1	wooden	antiphase	3PP
2	wooden	phase	3PP
3	wooden	antiphase	1PP
4	wooden	phase	1PP
5	human-like	antiphase	3PP
6	human-like	phase	3PP
7	human-like	antiphase	1PP
8	human-like	phase	1PP

Table S1. *List of experimental conditions and relative manipulations. 1PP: first-person perspective. 3PP: third-person perspective.*

N.	Ownership	Agency	Location	Nobody	Twobodies
1	15.4±27.4	43.6±39.9	18.8±32.7	18.9±24.5	19.0±30.5
2	29.6±34.3	59.4±40.0	23.5±35.2	19.7±30.8	22.6±32.4
3	52.9±37.3	64.7±34.1	85.3±21.0	17.6±29.7	16.2±26.6
4	55.4±35.8	60.7±32.5	86.8±16.4	17.2±30.3	14.8±28.5
5	22.4±31.7	43.3±38.4	17.8±32.2	23.7±30.3	18.0±29.1
6	30.8±32.9	65.3±36.8	21.3±32.4	23.4±30.0	18.3±28.6
7	60.8±35.6	52.5±34.7	88.6±16.8	7.35±17.5	10.6±23.2
8	65.3±34.8	71.1±30.6	85.0±20.5	7.92±19.3	9.36±20.6

Table S2. *Descriptive statistics (mean±standard deviation) of all the 8 conditions (from the less incongruent (1) to the most congruent (8)) for all the bodily self-consciousness constructs and control questions considered in this study (i.e., "body ownership", "body agency", "body location", "feeling of not having a body", "feeling of having more than one body").*

N.	CONSTRUCT	Statement	Statement Italian translation
1	Perceived body ownership	I felt that the virtual body/object was mine	Ho avuto la sensazione che il corpo/oggetto virtuale fosse il mio
2	Perceived body agency	I felt that I controlled the movements of the virtual body/object	Ho avuto la sensazione di controllare i movimenti del corpo/oggetto virtuale
3	Perceived body location	I felt that I was in the same place of the virtual body/object	Ho avuto la sensazione di occupare lo stesso luogo (spazio) del corpo/oggetto virtuale
4	Control question	I felt that I had no body	Ho avuto la sensazione di essere privo di corpo
5	Control question	I felt that I had more than one body	Ho avuto la sensazione di avere più di un corpo

Table S3. *Embodiment questionnaire on corporeal awareness. Replies are given by participants on separate digital visual-analogue scales(VAS)*

Task/questionnaire	Mean	Median	SD
Heartbeat Counting Task	0.51	0.57	0.25
Pneumoception Task	0.55	0.62	0.34
Interoceptive sensibility (MAIA-II questionnaire -"Noticing", "Body listening", "Attention regulation" subscales)	2.39	3	0.84
Body Image Concern (Body Uneasiness Test subscale)	1.41	1.33	0.94
Silhouette questionnaire	1.94	2	1.59
Menstrual Cycle Day	15.85	17	9.09

Table S4. *Descriptive statistics (Mean, median and standard deviation) of all the tasks indices and self-report measures analysed in the study.*

Fixed effects:	Estimate	Std. Error	t value	F value	Pr(>F)
(Intercept)	15.36857	5.95771	2.58		
**VISUAL.APPEARANCE**	**7.01529**	**6.14665**	**1.141**	**4.4743**	**0.035586**
**BREATH**	**14.27317**	**6.14665**	**2.322**	**5.7749**	**0.017126**
**PERSPECTIVE**	**37.56986**	**6.14665**	**6.112**	**122.6974**	**< 0.001**
Iacc	-2.44017	6.2687	-0.389	0.005	0.94383
Isen	4.12107	6.36023	0.648	0.0007	0.979394
VISUAL.APPEARANCE:BREATH	-5.86509	8.69268	-0.675	0.0941	0.759385
VISUAL.APPEARANCE:PERSPECTIVE	0.85631	8.69268	0.099	0.6191	0.432276
BREATH:PERSPECTIVE	-11.8903	8.69268	-1.368	1.6562	0.199538
VISUAL.APPEARANCE:Iacc	5.17452	6.46751	0.8	3.2524	0.072752
**VISUAL.APPEARANCE:Isen**	**-2.69837**	**6.56193**	**-0.411**	**8.6199**	**0.003696**
BREATH:Iacc	1.31381	6.46751	0.203	0.8891	0.346796
BREATH:Isen	-0.03643	6.56193	-0.006	0.298	0.585692
PERSPECTIVE:Iacc	1.67053	6.46751	0.258	0.0107	0.917697

PERSPECTIVE:Isen	-4.76912	6.56193	-0.727	1.8917	0.170474
VISUAL.APPEARANCE:BREATH:PERSPECTIVE	7.95997	12.29331	0.648	0.4193	0.518013
VISUAL.APPEARANCE:BREATH:Iacc	-3.84912	9.14644	-0.421	0.0048	0.944845
VISUAL.APPEARANCE:BREATH:Isen	-2.47376	9.27998	-0.267	1.5818	0.2099
VISUAL.APPEARANCE:PERSPECTIVE:Iacc	0.86677	9.14644	0.095	0.6375	0.425523
VISUAL.APPEARANCE:PERSPECTIVE:Isen	-5.61601	9.27998	-0.605	3.0156	0.083933
BREATH:PERSPECTIVE:Iacc	-9.17405	9.14644	-1.003	0.5686	0.451651
BREATH:PERSPECTIVE:Isen	11.90812	9.27998	1.283	0.8724	0.351364
VISUAL.APPEARANCE:BREATH:PERSPECTIVE:Iacc	8.59417	12.93502	0.664	0.4414	0.507156
VISUAL.APPEARANCE:BREATH:PERSPECTIVE:Isen	-11.5582	13.12387	-0.881	0.7756	0.379485

***Table S5**. Summary of perceived body ownership linear mixed effects model. Bold effects are statistically significant.*

Fixed effects:	Estimate	Std. Error	t value	F value	Pr(>F)
(Intercept)	43.6116	6.3905	6.824		
VISUAL.APPEARANCE	-0.3023	6.8457	-0.044	0.0788	0.77926
**BREATH**	**5.873**	**6.8457**	**2.3**	**14.6525**	**0.0007**
**PERSPECTIVE**	**2.0425**	**6.8457**	**3.074**	**7.4456**	**0.006898**
Iacc	-7.3576	6.724	-.094	0.5325	0.47204
Isen	0.765	6.8223	0.026	0.0007	0.978637
**VISUAL.APPEARANCE:BREATH**	**6.273**	**9.683**	**0.633**	**4.4086**	**0.036952**
VISUAL.APPEARANCE:PERSPECTIVE	-.8479	9.683	-.224	0.2768	0.599382
BREATH:PERSPECTIVE	-9.804	9.683	-2.046	2.8504	0.092837
VISUAL.APPEARANCE:Iacc	5.676	7.203	0.77	0.99	0.66775
VISUAL.APPEARANCE:Isen	2.9469	7.3082	0.403	0.0233	0.878938
BREATH:Iacc	5.7394	7.203	0.797	0.067	0.795833
BREATH:Isen	-.456	7.3082	-0.94	0.073	0.89543
PERSPECTIVE:Iacc	4.3438	7.203	0.603	0.4373	0.50935
PERSPECTIVE:Isen	-.4645	7.3082	-0.2	.4048	0.237254
VISUAL.APPEARANCE:BREATH:PERSPECTIVE	6.4928	3.694	.205	.45	0.229707
VISUAL.APPEARANCE:BREATH:Iacc	-0.9973	0.866	-.08	0.605	0.4375
VISUAL.APPEARANCE:BREATH:Isen	4.3734	0.3353	0.423	0.2674	0.60564
VISUAL.APPEARANCE:PERSPECTIVE:Iacc	-.5765	0.866	-0.55	0.2809	0.596672
VISUAL.APPEARANCE:PERSPECTIVE:Isen	-3.229	0.3353	-0.32	2.4255	0.20883
BREATH:PERSPECTIVE:Iacc	-7.747	0.866	-0.76	0.062	0.74482
BREATH:PERSPECTIVE:Isen	5.6486	0.3353	0.547	0.74	0.73222
VISUAL.APPEARANCE:BREATH:PERSPECTIVE:Iacc	0.7883	4.406	0.749	0.5608	0.45477
VISUAL.APPEARANCE:BREATH:PERSPECTIVE:Isen	-6.3053	4.664	-.6	.2444	0.26589

***Table S6**. Summary of perceived body agency linear mixed effects model. Bold effects are statistically significant.*

Fixed effects:	Estimate	Std. Error	t value	F value	Pr(>F)
(Intercept)	18.858	4.725	3.991		
VISUAL.APPEARANCE	-1.084	5.123	-0.212	0.0318	0.858711
BREATH	4.669	5.123	0.911	0.3551	0.551903
**PERSPECTIVE**	**66.429**	**5.123**	**12.966**	**664.9816**	**< 0.001**
Iacc	9.248	4.971	1.86	0.5874	0.4494

Isen	-6.707	5.044	-1.33	0.2571	0.615807
VISUAL.APPEARANCE:BREATH	-1.125	7.245	-0.155	0.3802	0.538162
VISUAL.APPEARANCE:PERSPECTIVE	4.414	7.245	0.609	0.2158	0.642717
BREATH:PERSPECTIVE	-3.127	7.245	-0.432	1.0148	0.314917
VISUAL.APPEARANCE:Iacc	-5.33	5.391	-0.989	0.0275	0.868433
VISUAL.APPEARANCE:Isen	8.645	5.469	1.581	0.0164	0.898254
BREATH:Iacc	-1.69	5.391	-0.314	0.0796	0.778097
BREATH:Isen	2.882	5.469	0.527	0.5155	0.473546
**PERSPECTIVE:Iacc**	**-12.023**	**5.391**	**-2.23**	**11.4978**	**< 0.001**
PERSPECTIVE:Isen	8.697	5.469	1.59	0.6439	0.423226
VISUAL.APPEARANCE:BREATH:PERSPECTIVE	-4.069	10.246	-0.397	0.1577	0.691708
VISUAL.APPEARANCE:BREATH:Iacc	5.949	7.623	0.78	0.2998	0.584614
VISUAL.APPEARANCE:BREATH:Isen	-7.744	7.735	-1.001	1.0231	0.312947
VISUAL.APPEARANCE:PERSPECTIVE:Iacc	6.816	7.623	0.894	0.5015	0.479608
VISUAL.APPEARANCE:PERSPECTIVE:Isen	-11.058	7.735	-1.43	2.6165	0.107261
BREATH:PERSPECTIVE:Iacc	1.95	7.623	0.256	0.0378	0.846005
BREATH:PERSPECTIVE:Isen	-4.158	7.735	-0.538	0.1266	0.722293
VISUAL.APPEARANCE:BREATH:PERSPECTIVE:Iacc	-5.996	10.781	-0.556	0.3093	0.578703
VISUAL.APPEARANCE:BREATH:PERSPECTIVE:Isen	4.423	10.939	0.404	0.1635	0.686347

Table S7*. Summary of perceived body location linear mixed effects model. Bold effects are statistically significant.*

Fixed effects:	Estimates	Std.Error	t value	F values	Pr(>F)
Intercept	15.01469	5.49557	2.732		
**VISUAL.APPEARANCE**	**7.50129**	**5.68072**	**1.32**	**17.332**	**<.0001**
**BREATH**	**14.36817**	**5.68072**	**2.529**	**21.1608**	**<.0001**
**PERSPECTIVE**	**37.8974**	**5.68072**	**6.671**	**311.191**	**< .0001**
SEX	1.73219	7.82466	0.221	0.38	0.539995
Iacc	-2.5794	6.07841	-0.424	0.0003	0.986182
Isen	3.66635	5.19049	0.706	0.0001	0.993079
VISUAL.APPEARANCE:BREATH	-6.03045	8.03375	-0.751	0.1209	0.728279
VISUAL.APPEARANCE:PERSPECTIVE	1.16377	8.03375	0.145	0.1337	0.714802
BREATH:PERSPECTIVE	-13.0638	8.03375	-1.626	0.3386	0.560973
VISUAL.APPEARANCE:SEX	2.31388	8.12459	0.285	0.2902	0.590393
BREATH:SEX	-5.81465	8.08827	-0.719	1.2047	0.273035
PERSPECTIVE:SEX	-0.14913	8.08827	-0.018	0.5446	0.460933
VISUAL.APPEARANCE:Iacc	5.46978	6.28319	0.871	1.0182	0.313533
**VISUAL.APPEARANCE:Isen**	**-2.40063**	**5.36535**	**-0.447**	**5.9314**	**0.015297**
**BREATH:Iacc**	**1.38878**	**6.28319**	**0.221**	**5.8719**	**0.015815**
BREATH:Isen	-0.03241	5.36535	-0.006	1.9052	0.168248
PERSPECTIVE:Iacc	1.76585	6.28319	0.281	2.0997	0.148093
**PERSPECTIVE:Isen**	**-4.24289**	**5.36535**	**-0.791**	**13.3517**	**0.000292**
SEX:Iacc	10.88189	8.20636	1.326	0.0175	0.895178
SEX:Isen	-0.64986	8.48465	-0.077	0.0019	0.965444
VISUAL.APPEARANCE:BREATH:PERSPECTIVE	9.07694	11.36144	0.799	0.6843	0.408597
VISUAL.APPEARANCE:BREATH:SEX	8.17774	11.46425	0.713	0.5129	0.474297
VISUAL.APPEARANCE:PERSPECTIVE:SEX	-6.06484	11.46425	-0.529	1.0873	0.297674

BREATH:PERSPECTIVE:SEX	14.71768	11.43855	1.287	2.322	0.12832
VISUAL.APPEARANCE:BREATH:Iacc	-4.06875	8.88577	-0.458	0.0064	0.936318
VISUAL.APPEARANCE:BREATH:Isen	-2.2008	7.58776	-0.29	0.2947	0.587512
VISUAL.APPEARANCE:PERSPECTIVE:Iacc	0.91623	8.88577	0.103	2.1181	0.14633
VISUAL.APPEARANCE:PERSPECTIVE:Isen	-4.99633	7.58776	-0.658	2.2339	0.13578
BREATH:PERSPECTIVE:Iacc	-9.69753	8.88577	-1.091	1.4096	0.235804
BREATH:PERSPECTIVE:Isen	10.59417	7.58776	1.396	0.0628	0.802302
VISUAL.APPEARANCE:SEX:Iacc	-10.7892	8.48356	-1.272	3.6015	0.058428
VISUAL.APPEARANCE:SEX:Isen	0.41569	8.77075	0.047	2.1699	0.141502
BREATH:SEX:Iacc	-5.97642	8.48283	-0.705	0.816	0.366866
BREATH:SEX:Isen	6.95663	8.7705	0.793	0.4271	0.513791
PERSPECTIVE:SEX:Iacc	-9.11061	8.48283	-1.074	1.6442	0.200472
PERSPECTIVE:SEX:Isen	-2.52545	8.7705	-0.288	3.3234	0.069027
VISUAL.APPEARANCE:BREATH:PERSPECTIVE:SEX	-4.75743	16.19473	-0.294	0.0863	0.769086
VISUAL.APPEARANCE:BREATH:PERSPECTIVE:Iacc	9.08456	12.56637	0.723	0.1614	0.688041
VISUAL.APPEARANCE:BREATH:PERSPECTIVE:Isen	-10.2829	10.73071	-0.958	0.3625	0.547433
VISUAL.APPEARANCE:BREATH:SEX:Iacc	4.05085	11.99705	0.338	0.0367	0.848159
VISUAL.APPEARANCE:BREATH:SEX:Isen	4.92114	12.40353	0.397	1.2801	0.258536
VISUAL.APPEARANCE:PERSPECTIVE:SEX:Iacc	7.10495	11.99705	0.592	0.0284	0.866323
VISUAL.APPEARANCE:PERSPECTIVE:SEX:Isen	2.16485	12.40353	0.175	0.6677	0.414312
BREATH:PERSPECTIVE:SEX:Iacc	5.91494	11.99654	0.493	0.0008	0.977553
BREATH:PERSPECTIVE:SEX:Isen	-18.1048	12.40335	-1.46	2.2319	0.135955
VISUAL.APPEARANCE:BREATH:PERSPECTIVE:SEX:Iacc	-11.3522	16.96603	-0.669	0.4477	0.503798
VISUAL.APPEARANCE:BREATH:PERSPECTIVE:SEX:Isen	10.00413	17.54112	0.57	0.3253	0.568769

Table S8*. Summary of perceived body ownership linear mixed effects model in male and female sample. Bold effects are statistically significant.*

Fixed effects	Estimates	Std.Error	t ratios	F values	Pr(>F)
Intercept	43.08077	5.82584	7.395		
VISUAL.APPEARANCE	-0.06394	6.26314	-0.01	0.2326	0.62987
**BREATH**	**16.28731**	**6.26314**	**2.601**	**29.1633**	**<.0001**
**PERSPECTIVE**	**21.41543**	**6.26314**	**3.419**	**16.2551**	**<.0001**
SEX	12.62608	8.2949	1.522	1.0962	0.29936
Iacc	-7.77748	6.4437	-1.207	2.437	0.12386
Isen	0.15703	5.50242	0.029	0.3058	0.58237
**VISUAL.APPEARANCE:BREATH**	**5.15398**	**8.85742**	**0.582**	**4.7085**	**0.03058**
VISUAL.APPEARANCE:PERSPECTIVE	-11.8186	8.85742	-1.334	0.0054	0.94142
BREATH:PERSPECTIVE	-20.6017	8.85742	-2.326	0.7478	0.38768
VISUAL.APPEARANCE:SEX	-1.87396	8.95745	-0.209	0.0001	0.99257
BREATH:SEX	-8.4353	8.91753	-0.946	0.2024	0.65306
PERSPECTIVE:SEX	-15.4426	8.91753	-1.732	0.0984	0.75387
VISUAL.APPEARANCE:Iacc	5.46249	6.92738	0.789	0.8252	0.36421
VISUAL.APPEARANCE:Isen	2.62175	5.91544	0.443	1.2955	0.2557
BREATH:Iacc	6.06689	6.92738	0.876	0.7222	0.39592
BREATH:Isen	-1.25937	5.91544	-0.213	0.2507	0.61686
PERSPECTIVE:Iacc	4.59167	6.92738	0.663	0.8178	0.36636

PERSPECTIVE:Isen	-1.30291	5.91544	-0.22	3.2828	0.07074
SEX:Iacc	2.09225	8.69954	0.241	0.1327	0.71694
SEX:Isen	1.60851	8.99455	0.179	0.2667	0.60751
VISUAL.APPEARANCE:BREATH:PERSPECTIVE	17.97361	12.52628	1.435	1.7232	0.19001
VISUAL.APPEARANCE:BREATH:SEX	-2.65524	12.63955	-0.21	0.996	0.31887
VISUAL.APPEARANCE:PERSPECTIVE:SEX	12.57433	12.63955	0.995	0.5012	0.47939
BREATH:PERSPECTIVE:SEX	21.76413	12.61129	1.726	3.0182	0.08308
VISUAL.APPEARANCE:BREATH:Iacc	-11.6248	9.79679	-1.187	1.4963	0.22195
VISUAL.APPEARANCE:BREATH:Isen	3.89084	8.3657	0.465	0.0029	0.9572
VISUAL.APPEARANCE:PERSPECTIVE:Iacc	-1.66651	9.79679	-0.17	2.7607	0.09737
VISUAL.APPEARANCE:PERSPECTIVE:Isen	-2.87279	8.3657	-0.343	2.1241	0.14576
BREATH:PERSPECTIVE:Iacc	-8.18349	9.79679	-0.835	1.186	0.27677
BREATH:PERSPECTIVE:Isen	5.02537	8.3657	0.601	0.0035	0.95272
VISUAL.APPEARANCE:SEX:Iacc	-8.44458	9.35334	-0.903	0.0381	0.84541
VISUAL.APPEARANCE:SEX:Isen	-10.0303	9.66998	-1.037	0.8707	0.3513
BREATH:SEX:Iacc	-5.01262	9.35255	-0.536	0.1832	0.66888
BREATH:SEX:Isen	-1.10386	9.6697	-0.114	0.4592	0.49838
PERSPECTIVE:SEX:Iacc	-7.34937	9.35255	-0.786	0.0297	0.86318
PERSPECTIVE:SEX:Isen	-5.41069	9.6697	-0.56	0.0475	0.82759
VISUAL.APPEARANCE:BREATH:PERSPECTIVE:SEX	-12.5087	17.85506	-0.701	0.4908	0.48397
VISUAL.APPEARANCE:BREATH:PERSPECTIVE:Iacc	11.40391	13.85475	0.823	0.0036	0.95191
VISUAL.APPEARANCE:BREATH:PERSPECTIVE:Isen	-14.5061	11.83088	-1.226	1.8577	0.17364
VISUAL.APPEARANCE:BREATH:SEX:Iacc	11.24486	13.22706	0.85	0.0019	0.96545
VISUAL.APPEARANCE:BREATH:SEX:Isen	4.87866	13.67522	0.357	0.4118	0.52141
VISUAL.APPEARANCE:PERSPECTIVE:SEX:Iacc	18.30854	13.22706	1.384	0.6377	0.42499
VISUAL.APPEARANCE:PERSPECTIVE:SEX:Isen	4.83224	13.67522	0.353	0.4057	0.52453
BREATH:PERSPECTIVE:SEX:Iacc	5.61694	13.2265	0.425	0.3118	0.57687
BREATH:PERSPECTIVE:SEX:Isen	2.55513	13.67502	0.187	0.1611	0.68831
VISUAL.APPEARANCE:BREATH:PERSPECTIVE:SEX:Iacc	-21.679	18.70549	-1.159	1.3432	0.24714
VISUAL.APPEARANCE:BREATH:PERSPECTIVE:SEX:Isen	2.65319	19.33954	0.137	0.0188	0.89095

Table S9. *Summary of perceived body agency linear mixed effects model in male and female sample. Bold effects are statistically significant.*

Fixed effects	Estimates	Std.error	t values	F values	Pr(>F)
Intercept	19.8093	4.5197	4.383		
VISUAL.APPEARANCE	-1.8417	5.0187	-0.367	1.2904	0.256638
BREATH	4.4228	5.0187	0.881	0.4887	0.484897
**PERSPECTIVE**	**65.1935**	**5.0187**	**12.99**	**1246.362**	**< .0001**
SEX	-2.1363	6.4352	-0.332	0.7508	0.389732
Iacc	9.7754	4.9991	1.955	0.2393	0.626566
Isen	-5.9669	4.2688	-1.398	0.2335	0.630749
VISUAL.APPEARANCE:BREATH	-0.3627	7.0975	-0.051	0.2803	0.596791
VISUAL.APPEARANCE:PERSPECTIVE	5.383	7.0975	0.758	0.6688	0.413951
BREATH:PERSPECTIVE	-2.8059	7.0975	-0.395	0.4942	0.482456
VISUAL.APPEARANCE:SEX	4.514	7.1776	0.629	2.0095	0.157071
BREATH:SEX	-4.1381	7.1457	-0.579	0.0541	0.816123

PERSPECTIVE:SEX	-6.7222	7.1457	-0.941	1.4745	0.225333
VISUAL.APPEARANCE:Iacc	-5.6346	5.551	-1.015	0.0232	0.879102
VISUAL.APPEARANCE:Isen	7.6914	4.7401	1.623	0.013	0.909204
BREATH:Iacc	-1.7865	5.551	-0.322	0.1759	0.675169
BREATH:Isen	2.5636	4.7401	0.541	0.0008	0.97703
**PERSPECTIVE:Iacc**	**-12.709**	**5.551**	**-2.289**	**17.5265**	**<.0001**
**PERSPECTIVE:Isen**	**7.7371**	**4.7401**	**1.632**	**6.2943**	**0.0124960**
SEX:Iacc	-10.7421	6.7491	-1.592	2.9401	0.0916697
SEX:Isen	10.1564	6.978	1.455	0.0348	0.852719
VISUAL.APPEARANCE:BREATH:PERSPECTIVE	-4.6885	10.0374	-0.467	0.3787	0.538645
VISUAL.APPEARANCE:BREATH:SEX	1.3402	10.1281	0.132	0.0517	0.82025
VISUAL.APPEARANCE:PERSPECTIVE:SEX	-0.5136	10.1281	-0.051	0.001	0.974665
BREATH:PERSPECTIVE:SEX	4.9851	10.1056	0.493	0.543	0.461616
VISUAL.APPEARANCE:BREATH:Iacc	6.2888	7.8503	0.801	0.7603	0.383743
VISUAL.APPEARANCE:BREATH:Isen	-6.8893	6.7035	-1.028	0.0752	0.784112
VISUAL.APPEARANCE:PERSPECTIVE:Iacc	7.2044	7.8503	0.918	0.9663	0.326176
VISUAL.APPEARANCE:PERSPECTIVE:Isen	-9.8382	6.7035	-1.468	1.9261	0.165937
BREATH:PERSPECTIVE:Iacc	2.061	7.8503	0.263	0.3725	0.541997
BREATH:PERSPECTIVE:Isen	-3.6992	6.7035	-0.552	0.5577	0.45562
VISUAL.APPEARANCE:SEX:Iacc	1.0002	7.4949	0.133	0.01	0.920395
VISUAL.APPEARANCE:SEX:Isen	-9.4662	7.7486	-1.222	0.0756	0.783531
BREATH:SEX:Iacc	1.4162	7.4943	0.189	0.0001	0.992264
BREATH:SEX:Isen	-1.3636	7.7484	-0.176	0.7621	0.383189
PERSPECTIVE:SEX:Iacc	5.5859	7.4943	0.745	0.9404	0.332734
**PERSPECTIVE:SEX:Isen**	**-17.0041**	**7.7484**	**-2.195**	**12.3665**	**<.0001**
VISUAL.APPEARANCE:BREATH:PERSPECTIVE:SEX	0.5726	14.3074	0.04	0.0016	0.968094
VISUAL.APPEARANCE:BREATH:PERSPECTIVE:Iacc	-6.338	11.1019	-0.571	0.5377	0.463812
VISUAL.APPEARANCE:BREATH:PERSPECTIVE:Isen	3.9352	9.4802	0.415	0.0004	0.983506
VISUAL.APPEARANCE:BREATH:SEX:Iacc	-0.5473	10.599	-0.052	0.0016	0.968592
VISUAL.APPEARANCE:BREATH:SEX:Isen	11.8147	10.9581	1.078	0.9924	0.31973
VISUAL.APPEARANCE:PERSPECTIVE:SEX:Iacc	-1.5462	10.599	-0.146	0.0088	0.925241
VISUAL.APPEARANCE:PERSPECTIVE:SEX:Isen	9.0831	10.9581	0.829	0.4143	0.520134
BREATH:PERSPECTIVE:SEX:Iacc	-3.2004	10.5985	-0.302	0.099	0.753221
BREATH:PERSPECTIVE:SEX:Isen	1.7723	10.9579	0.162	0.0899	0.764456
VISUAL.APPEARANCE:BREATH:PERSPECTIVE:SEX:Iacc	1.6851	14.9889	0.112	0.0126	0.91054
VISUAL.APPEARANCE:BREATH:PERSPECTIVE:SEX:Isen	-8.191	15.497	-0.529	0.2794	0.597398

Table S10. *Summary of perceived body location linear mixed effects model in male and female sample. Bold effects are statistically significant.*

	Frequency	Amplitude	Age	Smoke/Day	Sport/Week
**Mean**	16.9575758	28.374754	24.40816	2.693878	1.489796
**St.Dev.**	3.621725	5.307017	3.135272	4.606174	2.255756
**Min**	9.2	21.2892	19	0	0
**Max**	24.6	44.82	35	20	10

Table S11. *Descriptive statistics of breathing tracks used as 'self' stimuli in the Pneumoception task. Frequency (cy./min.): participants' respiratory frequency (breaths per minute). Amplitude (db): participants' respiratory amplitude (decibels). Smoke (cigs./day): participants' average daily number of cigarettes. Sport (hrs./wk.): participants' average weekly hours devoted to sports.*

Supplemental figures S1-S4

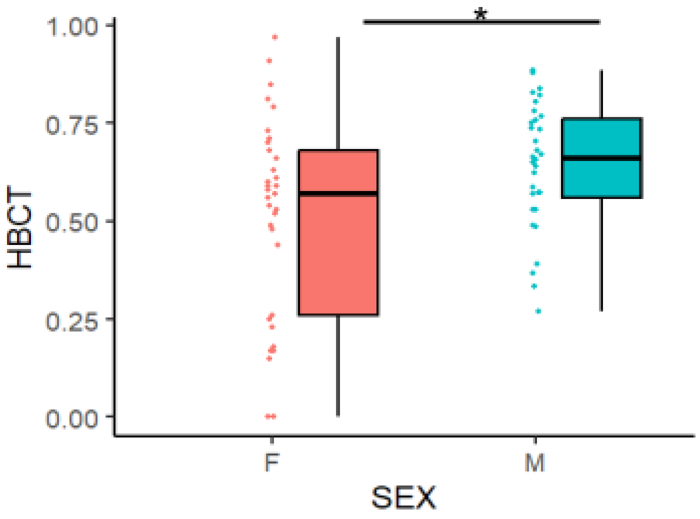

Figure S1. Differences between men and women in interoceptive accuracy measured through the Heartbeat Counting Task (HBCT). Significance: '' p<.05. Male sample data were taken from Monti and collaborators dataset [11].*

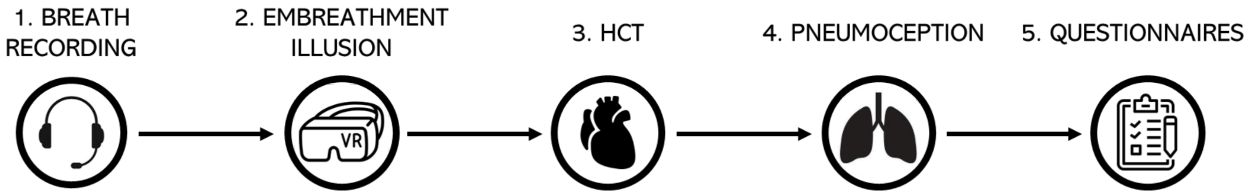

Figure S2. Visual description of all the experimental paradigms used in the present study

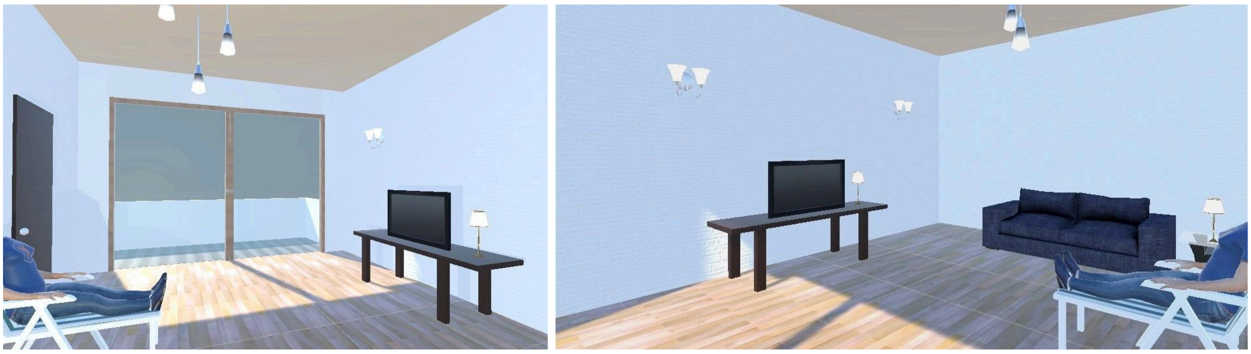

Figure S3. Picture of the virtual living room used in the experiment

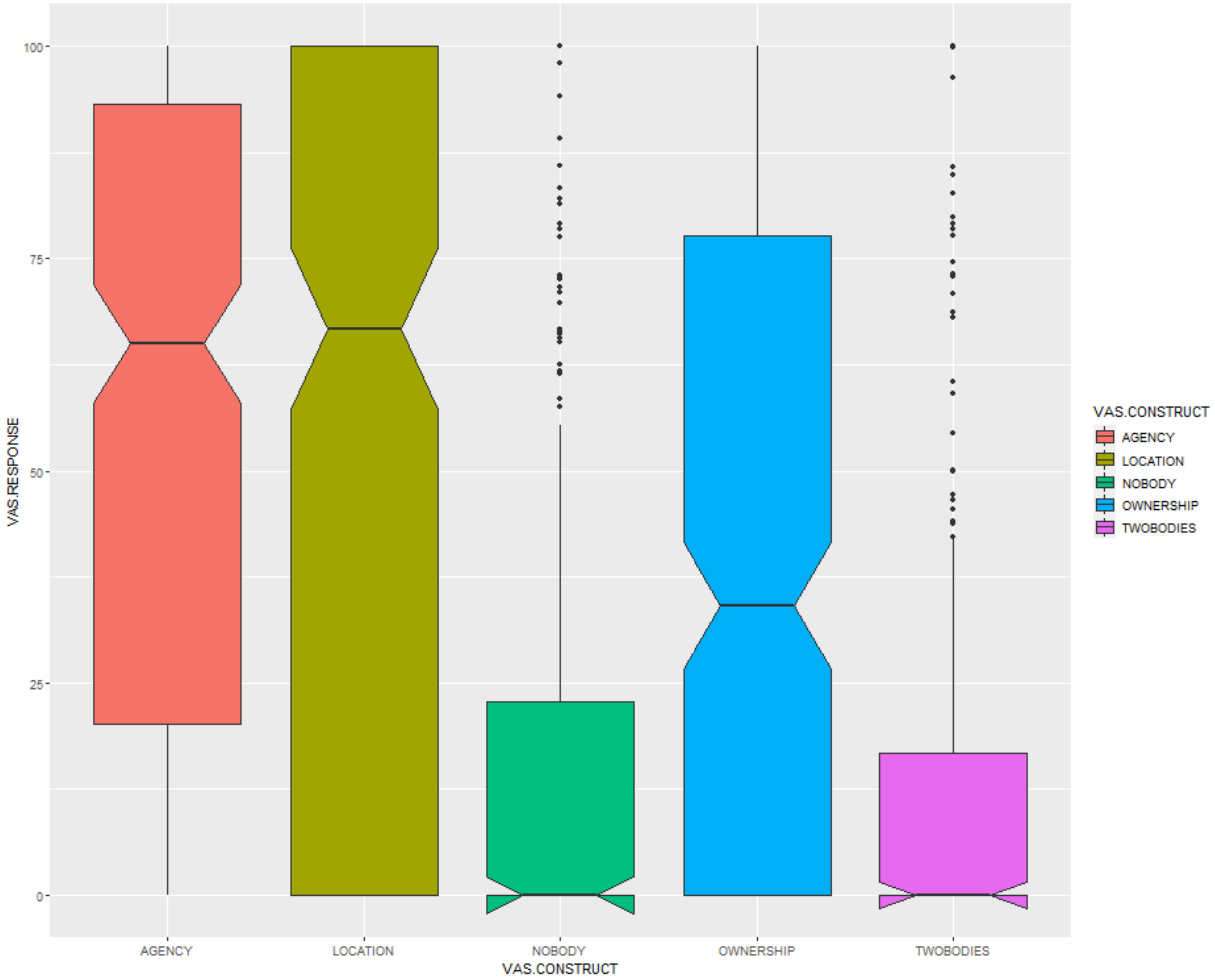

Figure S4. *Boxplot of visual analogue scale (VAS) responses to experimental questions (Ownership, Agency, Location) and control questions, (Nobody and Two-Body), See Table S3.*

Detailed experimental protocols

Virtual reality apparatus and stimuli

Please see above a detailed description of the paradigms used for the experiment (see also Fig. S2). The immersive virtual reality scenario consisted of life-size three-dimensional avatars lying on a deck chair in a customized living room. Two types of avatars were designed with MakeHuman 1.2.0 software (MakeHuman™) and 3DS Max 2015: the 'human-like' avatar closely looked like a human body, while in the 'wooden' avatar each human body part was replaced with a wooden, squared box preserving the length and the width of the original "human" part. Each type of avatar came in ten different sizes, allowing participants to tailor the avatar to the size of their real body. The virtual deck chair was designed with 3DS Max 2015 matching the physical dimensions and appearance of a real deck chair. The virtual living room was built similarly to a real one and included a door, a sofa, a flat TV screen, several lamps and a door window looking out onto a balcony. Walls, floors, doors, furniture, and windows were designed with Unity 5.6.0s3 (See Fig. S3). The virtual environment was live broadcast in Unity 5.6.0s3 on an HTC Vive head-mounted display (field of view: 110°, resolution: 2160×1200 px, resolution per eye: 1080×1200 px, aspect ratio: 9/5, refresh rate: 90 Hz; HTC Corp.). The Vive accelerometer, gyroscope, Lighthouse laser tracking system, and front-facing cameras automatically detected head movements and adjusted the computer-generated image accordingly. A Vive controller was tied to a belt worn by participants to track their breathing and live map the corresponding belly movements onto the avatars using a customised Unity 5.6.0s3 script.

Interoception tasks

In addition to the virtual bodily illusion, the experiment also included three interoception tests:

1. The heartbeat counting task (HBCT[1]), in which participants are required to report the number of heartbeats they perceive in four different time windows (i.e., 25s; 35s; 45s and 100s) while their cardiac activity is recorded through a standard electrocardiogram (ECG). Electrocardiographic data were recorded by mean of three pre-gelled, disposable Ag/AgCl 50 mm electrodes arranged in a bipolar lead II configuration and connected to an ADInstruments PowerLab 8/30 data acquisition system, coupled with an ADInstruments BioAmp biological signal amplifier (ADInstruments). Individual electrocardiograms were processed on LabChart 7.3.8 (ADInstruments) to find QRS complexes and thus compute heart rates in each time interval. Subjective heartbeat estimates were collected with E-Prime 2.0 (Psychology Software Tools, Inc.).
2. A 'Pneumoception' task[2] assessing participants' ability to discriminate between the sound of their breath and someone else's. At the beginning of the experimental session, breath sounds were recorded with Audacity 3.2.3 software through a Sennheiser PC5 CHAT noise-cancelling microphone placed in the area between the nostrils and the upper lip (impedance: 32Ω, frequency response: 42-17000 Hz, pick-up pattern: unidirectional; Sennheiser electronic GmbH & Co. KG). To make the participants breathe as spontaneously as possible, a cover story was used saying that the experimenters would record the pronunciation of unusual words before and after a period of relaxation. Thus, participants pronounced two lists of words, and their naturally occurring breaths were covertly recorded in a long pause between the two lists. Breath sound tapes were then edited using Praat 6.0.29 software[3] and cut into 23s artefact-free tracks. For each track, the mean frequency and amplitude were computed. Then, a custom-made MATLAB R2019a script (The MathWorks, Inc.) matched the breath recording of a participant (self-tracks) with another's (nonself-tracks). To prevent ceiling effects, the nonself-tracks frequency and amplitude could be at most 25% higher or lower than the self-tracks'. Nonself-tracks were randomly taken from a database of previously collected recordings ($N = 16$, all females), updated with

a new track after each experimental session (Total breath tracks: 49, all females). All tapes (13 self-tracks and 13 nonself-tracks) were finally played in random order on the MATLAB Psychophysics Toolbox[4–6] to record reaction time and accuracy data: at the end of each tape, participants were asked to press the "i" keyboard key when the recording they heard was their own breathing sound, and the "o" keyboard key when the recording they heard was someone else's.

This Pneumoception task was primarily chosen to replicate the results found by Monti and colleagues in a previous study[2], testing whether they held also in a female sample. Furthermore, we decided to implement this task and not a respiratory load task to make sure that the experience was as ecological and spontaneous as possible by simulating everyday breathing.

3. The Italian version of the Multidimensional Assessment of Interoceptive Awareness (MAIA) 2nd version was used to assess participants' interoceptive sensibility. This questionnaire consists of 37 items measuring the degree to which someone is aware of their physiological condition with a 5-point Likert scale[7,8].

Self-report measures of Body Image Dissatisfaction

Two measures of body image dissatisfaction were collected during the experiment:

1. Silhouette Rating Scale[9]. This test is composed of two identical sets of stimuli including nine feminine silhouettes, varying for body dimensions (width of body parts) and shape: the first one is the thinnest and the ninth is the largest. Each set of stimuli is accompanied by two different instructions. The first set of instructions is: "Please, observe the nine figures and select the one that, from 1 to 9, you perceive as most accurately depicting your current body size". While the second is: "Please, observe the nine figures and select the one that, from 1 to 9, you perceive as most accurately depicting how you would like to be". The

response given to the first set of stimuli is considered a measure of one's current body shape and size; the response given to the second set of stimuli is considered a measure of one's ideal body shape and size. The discrepancy in absolute value between the second and the first response (ideal - current body shape and size ratings) is considered a measure of body dissatisfaction, thus the higher it is the higher body dissatisfaction.

2. Body Image Concern subscale of Body Uniseaness Test-A[10] consists of 6-items Likert scale (from 0 to 6). Its score is calculated from the average of items 3,4,6,12,15,22,23,25, and 34. The higher is the score the higher is body image concerns.

Experimental procedure

After reading and signing the informed consent, the breath sound of the participants was recorded following the above-mentioned procedure. Then participants lay down on a deck chair wearing the HTC Vive and immersed themselves in the virtual reality scenario. Here, during the calibration phase, participants saw a human-like avatar in a first-person perspective (1PP), and they were asked to guide the experimenter in order to customize the size and the viewpoint of the avatar so that the virtual body was perceived as corresponding to the real body. Then they were asked to focus on a virtual lamp while a virtual panel temporarily screened them off from the virtual room, blocking the view of the virtual body. Meanwhile, the experimenters fastened a belt tied to an HTC Vive controller to the participant's abdomen to detect participants' online breath movements. At the end of the calibration phase, unbeknownst to the participant, the experimenter started a Unity script that mapped the participant's breathing pattern onto the virtual belly of the avatar in real time. Then, the experimenter observed the respiration-induced motion of both the virtual and the real belly to check that there was no detectable delay between the real and the virtual respiratory movements and that the moving virtual belly was seamlessly integrated into the virtual body mesh. In 2 participants, an error in the moving virtual belly occurred, so they were discarded from the analyses. To avoid breath-related habituation and priming effects, the breathing script was not activated during the

calibration phase. Moreover, the order of experimental conditions was counterbalanced across participants with a Williams design, i.e., a generalised Latin square that is also balanced for the estimation of first-order carryover effects[11]. Participants were instructed, at the onset of each trial, to focus either on the torso of the body or the virtual object positioned in front of them or to their right. They were then asked to minimize movement on the deck chair. At the end of each trial, participants had to answer a 5-item embodiment questionnaire using a visual analogue scale (VAS) indicating how much they agreed with each statement from 0 ("I did not have that feeling at all") to 100 ("I perceived a strong feeling of that sort") through a joystick-controlled cursor (Table S2). A short training session helped them familiarize with the response method. Each one of the eight experimental conditions lasted two minutes and showed an avatar whose physical appearance, perspective, and breathing pattern could either be congruent or incongruent with the real-world participant. Thus, the avatar could either be a 'human-like' or 'wooden' virtual character; it could be seen either from the first- or third-person perspective (in the latter case, the avatar was displayed at a distance of ≈ 40 cm to the participant's right side, and a panel covered the part above the neck); and it breathed either in-phase with the participant, or following an opposite breathing pattern, i.e., it inhaled when the participant exhaled, and vice versa (Table S1).

Data analysis

Corporeal awareness ratings during the 'embreathment' illusion. The primary goal of the present study was to replicate the results of Monti and collaborators[2] in a female sample. Specifically, we aimed to investigate the role of breathing, visual appearance, and spatial perspective on corporeal awareness. Hence, we used RStudio 2022.02.3 and the R lme4 package[12] to perform a linear mixed-effects analysis assessing how much ratings of perceived body ownership, agency, and location, collected through Visual Analogue Scales (VAS), changed when manipulating visual, respiratory, and spatial features of the virtual body to make them congruent or incongruent with those of the real body. Moreover, we tested whether such rating changes were influenced by

interoceptive sensibility and accuracy scores. Interoceptive accuracy was computed as the mean of Heartbeat Counting Task scores (measured by comparing objective and estimated heartbeat counts in the Heartbeat Counting Task[1]) and Pneumoception task scores (calculated with the non-parametric index of sensitivity *A*[13,14]). Interoceptive sensibility was assessed through three MAIA subscales of interest – *noticing*, *attention regulation*, and *body listening*[8]. Specifically, since the MAIA II questionnaire consists of eight subscales but does not have a single score to assess interoceptive sensibility (for correlations between MAIA-II subscales, see Table S12), we selected three subscales of interest and created a single index. Specifically, the study aimed to explore: participants' ability to listen to their body signals (as measured by the "Body Listening" subscale), as we induced an illusion based on a body signal (i.e., breath); participants' tendency to notice body sensations (i.e., in our case breath) (as measured by the "Noticing" subscale); participants' ability to sustain and control attention toward their body sensations (as measured by the "Attentional Regulation" subscale), to see if they could actually maintain focus on the avatar's breath during the illusion.

Correlation Matrix

		Noticing	Not-Distracting	Not-Worrying	Attentional Regulation	Emotional Awareness	Self Regulation	Body Listening	Trusting
Noticing	Pearson's r	—							
	df	—							
	p-value	—							
Not-Distracting	Pearson's r	0.067	—						
	df	31	—						
	p-value	0.710	—						
Not-Worrying	Pearson's r	0.128	0.063	—					
	df	31	31	—					
	p-value	0.479	0.726	—					
Attentional Regulation	Pearson's r	0.743 ***	0.159	0.315	—				
	df	31	31	31	—				
	p-value	< .001	0.377	0.074	—				
Emotional Awareness	Pearson's r	0.669 ***	0.060	0.328	0.669 ***	—			
	df	31	31	31	31	—			
	p-value	< .001	0.738	0.063	< .001	—			
Self Regulation	Pearson's r	0.479 **	0.137	0.285	0.731 ***	0.659 ***	—		
	df	31	31	31	31	31	—		
	p-value	0.005	0.446	0.108	< .001	< .001	—		
Body Listening	Pearson's r	0.475 **	0.139	0.351 *	0.621 ***	0.706 ***	0.717 ***	—	
	df	31	31	31	31	31	31	—	
	p-value	0.005	0.442	0.045	< .001	< .001	< .001	—	
Trusting	Pearson's r	0.405 *	0.061	0.275	0.551 ***	0.706 ***	0.707 ***	0.791 ***	—
	df	31	31	31	31	31	31	31	—
	p-value	0.020	0.736	0.121	< .001	< .001	< .001	< .001	—

Note. * p < .05, ** p < .01, *** p < .001

Table S12. *Correlation Matrix of all the eight MAIA-II subscales*

This approach allowed us to sidestep the multicollinearity assumption, as the three subscales were highly correlated with each other (Attentional regulation - Noticing: R = .74, $p < 0.001$; Noticing - Body Listening: R = .48, $p < 0.01$; Body Listening - Attentional Regulation: R = .62, $p < 0.001$). Moreover, being consistent with the analyses run by Monti and colleagues (2020), this methodology facilitated the comparison of interoceptive sensibility scores between men and women.

We built three linear mixed models following the analysis run by Monti and colleagues[2]. The dependent variables in the linear mixed models were VAS scores of body ownership, agency, and location. All models considered appearance (two levels: human-like and wooden), breathing (two

levels: phase and antiphase), and perspective (two levels: first- and third-person perspective) as fixed effects. These fixed effects were tested for interactions with each other, as well as with the interoceptive sensibility (*isen*) and accuracy (*iacc*) scores using *emmeans* package[15]. Finally, as random effects, the models included by-subject intercepts and fixed slopes:

$$p.ownership \sim app * breath * persp * (isen + iacc) + (1 | subj) \qquad (3.1)$$

$$p.agency \sim app * breath * persp * (isen + iacc) + (1 | subj) \qquad (3.2)$$

$$p.location \sim app * breath * persp * (isen + iacc) + (1 | subj) \qquad (3.3)$$

Corporeal awareness ratings during the 'embreathment' illusion in males and females. In order to investigate the role of breathing, visual appearance, and spatial perspective on corporeal awareness in both males and females, we ran three different linear mixed models as described above, with the addition of Sex as a factor (2 levels: female and male).

$$p.ownership \sim app * breath * persp * sex * (isen + iacc) + (1 | subj) \qquad (3.4)$$

$$p.agency \sim app * breath * persp * sex * (isen + iacc) + (1 | subj) \qquad (3.5)$$

$$p.location \sim app * breath * persp * sex * (isen + iacc) + (1 | subj) \qquad (3.6)$$

The relative importance of each effect was gauged by comparing its standardised regression coefficient with the others'[15,16].

All models specified in equations 3.1, 3.2, 3.3, 3.4, 3.5, and 3.6 were tested for linearity, absence of collinearity, homoscedasticity, normality of residuals, and absence of influential data points. *p-values* were computed through Type III Wald chi-square tests performed with the *Anova* function of the R *car* package[17]. Marginal and conditional R^2 goodness-of-fit measures of each mixed-effects model[18,19] were calculated with the R *MuMIn* package[20].

Data availability

The datasets generated and analysed during the current study and supplementary materials are available on the Open Science Framework repository (https://osf.io/s8xne/)

Additional datasets and data analysis

Experimental controls

Tests of statistical assumptions of linear mixed-effects models. For all linear mixed-effects models that were included in the final data analysis, visual inspection of residual plots did not reveal any obvious deviation from the standard assumptions of linearity, homoscedasticity, and normality of residuals. Condition indices of the matrix of the independent variables[21] showed no collinearity problem (all indices < 30). DFBETAS values[22,23] confirmed that there were no influential data points (all values < 2).

Specificity of experimental manipulations. Figure S4 summarises the main descriptive statistics of VAS ratings for experimental and control questions. Since the notches of the boxes relative to the experimental questions never overlap with the notches of the control questions boxes, there is strong evidence that the experimental medians differ from the control medians[24]. Across conditions, the average perceived body ownership, agency, and location were 41.57 (SD = 37.90, range: 0-100), 57.58 (SD = 36.81, range: 0-100), and 53.38 (SD = 42.50, range: 0-100), respectively.

Analyses of control items. Results of the analyses run on the control items revealed only a main effect of the perspective on the "Nobody" ($F(1,210)=9.8717$; p-value=0.001921) and "Two-bodies" ($F(1,210)=6.5957$; p-value= 0.01092) questions. Specifically, when in a third-person perspective, participants felt more that they had no body and that they had more than one body compared to when they were in the first-person perspective. This could be due to the fact that in the third-person perspective, participants could have experienced a dissociation from their own body, resulting in the perception of not having a body or, alternatively of having more than one body. These results are in

line with studies showing that the sense of embodiment is lower when the avatar is located and observed from the third-person perspective [25-27]. Finally, it is worth noting that no effect of visual appearance or breathing manipulations emerged by analysing the two control questions, confirming the specificity of the effect of these manipulations on the selected facets of the bodily self-consciousness namely sense of body ownership, agency and location.

Interoception indices results

Heartbeat counting task. The average score in the Heartbeat counting task (HCT) score was 0.52 (SD = 0.56, range: 0-0.97).

Pneumoception task. According to binomial distribution probabilities, participants who performed above chance should have a score equal to or above 69.23% (18 hits or correct rejections out of 26 trials, p = .038). 10 participants out of 33 (30.3%) scored above this threshold, reliably discriminating their own breath from others'. The average sensitivity index was 0.56 (SD = 0.34, range: 0-1). Metacognitive judgments of performance had a mean value of 54.03 (SD = 23.38, range: 0-97).

MAIA. The average scores of the three MAIA subscales of interest - *noticing, attention regulation and body listening* were 3.23 (SD = 0.92, range: 1.75-5), 2.62 (SD = 0.98, range: 0.71-4.57), and 2.94 (SD = 1.06, range: 0.33-4.67), respectively.

Interoceptive sensibility. The average *isen* score was 2.93 (SD = 0.85, range: 1.58-4.55).

Supplemental references

1. Schandry, R. Heart beat perception and emotional experience. *Psychophysiology* **18**, 483–488 (1981).

2. Monti, A., Porciello, G., Tieri, G. & Aglioti, S. M. The “embreathment” illusion highlights the role of breathing in corporeal awareness. *J Neurophysiol* (2020).

3. Boersma, P. & Weenink, D. Praat: doing phonetics by computer [software]. (2017).

4. Brainard D. H. he Psychophysics Toolbox. *Spat Vis* **10**, 433–436 (1997).

5. Kleiner, M., Brainard, D. & Pelli, D. What’s new in Psychtoolbox-3? (2007).

6. Pelli, D. G. & Vision, S. The VideoToolbox software for visual psychophysics: Transforming numbers into movies. *Spat Vis* **10**, 437–442 (1997).

7. Calì, G., Ambrosini, E., Picconi, L., Mehling, W. E. & Committeri, G. Investigating the relationship between interoceptive accuracy, interoceptive awareness, and emotional susceptibility. *Front Psychol* **6**, 1202 (2015).

8. Mehling, W. E., Acree, M., Stewart, A., Silas, J. & Jones, A. The multidimensional assessment of interoceptive awareness, version 2 (MAIA-2). *PLoS One* **13**, e0208034 (2018).

9. Lombardo, C., Cerolini, S., Esposito, R. M. & Lucidi, F. Psychometric properties of a Silhouette Rating Scale assessing current and ideal body size and body dissatisfaction in adults. *Eating and Weight Disorders-Studies on Anorexia, Bulimia and Obesity* **27**, 1089–1097 (2022).

10. Cuzzolaro, M., Vetrone, G., Marano, G. & Garfinkel, P. The Body Uneasiness Test (BUT): development and validation of a new body image assessment scale. *Eating and Weight Disorders-Studies on Anorexia, Bulimia and Obesity* **11**, 1–13 (2006).

11. Williams, E. J. Experimental designs balanced for the estimation of residual effects of treatments. *Aust J Chem* **2**, 149–168 (1949).

12. Bates, D., Mächler, M., Bolker, B. & Walker, S. Fitting Linear Mixed-E ects Models Using lme. *Journal of Statistical Software ()*.

13. Pollack, I. & Norman, D. A. A non-parametric analysis of recognition experiments. *Psychon Sci* **1**, 125–126 (1964).

14. Mueller, S. T. & Zhang, J. A note on ROC analysis and non-parametric estimate of sensitivity. (2005).

15. Lenth, R., Singmann, H., Love, J., Buerkner, P. & Herve, M. Package ‘emmeans’. Preprint at (2019).

16. Darlington, R. B. *Regression and linear models*. (McGraw-Hill Companies, 1990).

17. Johnson, J. W. & LeBreton, J. M. History and use of relative importance indices in organizational research. *Organ Res Methods* **7**, 238–257 (2004).

18. Fox, J. & Weisberg, S. Multivariate linear models in R. *An R Companion to Applied Regression. Los Angeles: Thousand Oaks* (2011).

19. Johnson, P. C. D. Extension of Nakagawa & Schielzeth's R2GLMM to random slopes models. *Methods Ecol Evol* **5**, 944–946 (2014).

20. Nakagawa, S. & Schielzeth, H. A general and simple method for obtaining R2 from generalized linear mixed‐effects models. *Methods Ecol Evol* **4**, 133–142 (2013).

21. Barton, K. MuMIn: multi-model inference. R package. *Cran-R* **1**, 289–290 (2018).

22. Belsley, D. A., Kuh, E. & Welsch, R. E. *Regression diagnostics: Identifying influential data and sources of collinearity*. (John Wiley & Sons, 2005).

23. Nieuwenhuis, R., Te Grotenhuis, H. F. & Pelzer, B. J. Influence. ME: tools for detecting influential data in mixed effects models. (2012).

24. McGill, R., Tukey, J. W. & Larsen, W. A. Variations of box plots. *Am Stat* **32**, 12–16 (1978).

25. Maselli, A., & Slater, M. (2013). The building blocks of the full body ownership illusion. Frontiers in Human Neuroscience, 7, 83.

26. Petkova, V. I., Khoshnevis, M., & Ehrsson, H. H. (2011). The perspective matters! Multisensory integration in ego-centric reference frames determines full-body ownership. Frontiers in Psychology, 2, 35.

27. Slater, M., Spanlang, B., Sanchez-Vives, M. V, & Blanke, O. (2010). First person experience of body transfer in virtual reality. PloS One, 5(5), e10564.
